# Accelerating countermeasure candidate discovery for A-series chemical warfare agent exposure

**DOI:** 10.1073/pnas.2512471122

**Published:** 2025-07-17

**Authors:** Nicolás M. Morato, Katelyn E. Mason, Todd H. Corzett, Carlos A. Valdez, Teneile M. Alfaro, Saphon Hok, R. Graham Cooks, Brian P. Mayer

**Affiliations:** ^a^Purdue Institute for Cancer Research, Purdue University, West Lafayette, IN 47907; ^b^Forensic Science Center, Global Security Principal Directorate, Lawrence Livermore National Laboratory, Livermore, CA 94550; ^c^Department of Chemistry, Purdue University, West Lafayette, IN 47907

**Keywords:** acetylcholinesterase, enzyme inhibition, mass spectrometry, medical countermeasures, high throughput reaction screening

## Abstract

The recent alleged use of A-series chemical warfare agents (CWAs) highlights the urgent need to better understand their inhibition of cholinesterase enzymes and the reported shortcomings of traditional oxime countermeasures. Here, using high-throughput (HT) mass spectrometry (MS) technologies, we characterized the largely unknown inhibition kinetics of A-series CWAs on human acetylcholinesterase (*h*AChE) and its reactivation by oximes, achieving label-free quantitation at rates of up to 7,000 reactions per hour. Our findings indicate i) A-series agents exhibit inhibitory potencies similar to traditional CWAs like sarin and VX, and ii) bipyridinium-based oximes can reactivate A-series-adducted *h*AChE in vitro, challenging prior reports on oxime efficacy. These results underscore the need for continued exploration of countermeasure candidates against A-series CWAs and demonstrate the potential of HT-MS for rapidly and safely characterizing emerging toxic chemicals.

## Significance

This study uses high-throughput mass spectrometry to address the urgent need for countermeasures against A-series chemical warfare agents (“Novichoks”). Using a surrogate- and label-free approach, we provide the first quantitative report on the in vitro potency of this group of agents as inhibitors of human acetylcholinesterase, demonstrating that they are on par with traditional agents. We also confirm the high stability of these enzyme-agent adducts while revealing the potential of bispyridinium-based oximes as scaffolds for new countermeasure candidates. By automating research with authentic toxic materials using ultrafast (>1 sample/s) mass spectrometry platforms, this work demonstrates a decentralized capability for safe, large-scale research on emerging chemical threats, enabling robust insights into their bioactivities and greatly accelerating the discovery of countermeasure candidates.

**Fig. 1.**
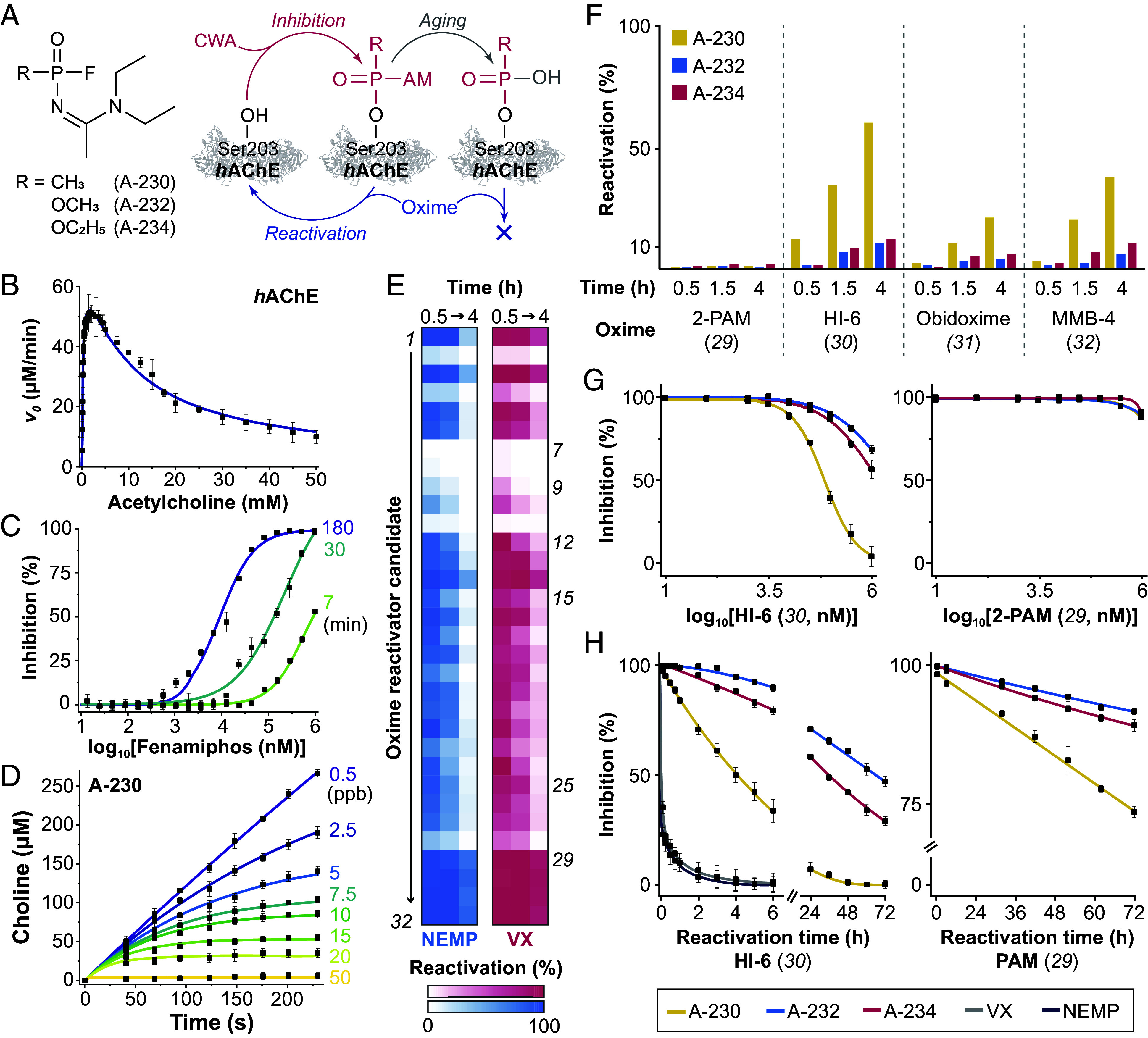
Characterization of inhibition and reactivation behaviors of amidine A-series CWAs using label-free (U)HT-MS. (*A*) Structures of the three A-series agents studied as well as their general reaction with the Ser203 residue in the active site of *h*AChE. AM represents the amidine side chain shared by the agents. (*B*) Representative data in the validation of the label-free MS methodology for kinetic characterization of ChEs. The results for the study of *h*AChE via UHT-DESI-MS using acetylcholine as substrate are shown, including the nonlinear fitting of the observed substrate inhibition behavior. (*C*) Representative data in the characterization of *h*AChE inhibition by phosphoramidate pesticides via UHT-DESI-MS. Three out of eleven preincubation timepoints (min) of fenamiphos with *h*AChE are shown. (*D*) Representative data in the characterization of *h*AChE inhibition by the amidine A-series agents via HT-ESI-MS. Results from the coincubation of *h*AChE with A-230 at different concentrations (ppb) are shown. (*E*) UHT-DESI-MS screening of an oxime candidate library (100 µM; see *SI Appendix*, Table S4 for structures) against VX- and NEMP-adducted *h*AChE. Three reactivation times were evaluated (0.5, 1, and 4 h). (*F*) Reactivation of A-series-exposed *h*AChE shown over ≤4 h for oximes *29*-*32* (100 µM). (*G*) Confirmatory dose–response curves for A-series-adducted *h*AChE (4 h). (*H*) Longer reactivation time (≤72 h) results for 2-PAM and HI-6. In all cases, data points represent averages and error bars indicate SD.

Recent high-profile attempted assassinations have drawn increased attention to nontraditional, A-series chemical warfare agents (CWAs), commonly branded as “Novichoks” (1–3). Like traditional CWAs (e.g., sarin, VX), A-series agents are organophosphorus compounds that act primarily as disruptors of neurotransmission via covalent inhibition of acetylcholinesterase (AChE), a key enzyme involved in the termination of cholinergic nerve impulses via rapid hydrolysis of acetylcholine into choline at the neuron synapses (4, 5). The generation of inactive AChE adducts through stable phosphoester bonding of the agent to a serine residue in the active site of the enzyme leads to accumulation of acetylcholine and nerve receptor overstimulation, which can in turn result in muscular paralysis and death by asphyxia or cardiac arrest (6, 7). However, the inactive AChE–CWA complexes may be reactivated by an appropriate countermeasure, such as an oxime, which reacts with the phosphoserine adduct via a nucleophilic attack, restoring the original serine residue, thus reinstating enzyme activity (8, 9). Note that reactivation is possible only if the adduct has not undergone aging, a spontaneous hydrolytic reaction that generates a dead-end, permanently inactive species (6, 7). The biochemical interactions of CWAs and AChE are summarized in Fig. 1*A*.

Although A-series agents share these mechanistic similarities with traditional CWAs, publicly available data on their potency as AChE inhibitors and the efficacy of conventional oxime countermeasures against them are still notably scarce, as are studies on their aging. Most available reports are anecdotal (2, 3, 10–12), rely on surrogate material (13, 14), or are based on computational work lacking experimental validation (15–17). These limitations, along with conflicting interpretations, likely result from a scarcity of extensive chemical and toxicological data, with the structures of these emergent agents being substantiated only after their addition to the Chemical Weapons Convention (CWC) in 2020 (18). Additionally, restricted access to authentic CWA material (14) and the absence of specialized facilities for large-scale in vitro studies have further impeded advancements in understanding and countering these threats.

Here, we extend the paradigm of counter-CWA research by employing automated high-throughput (HT) mass spectrometry (MS) technologies to provide insight into AChE inhibition by A-series CWAs while demonstrating safe, collaborative workflows for rapid countermeasure candidate discovery. To achieve this, we leveraged recent advances in drug discovery, where HT (≥10^3^ samples per day) and ultra-HT (UHT, ≥10^5^ samples per day) MS have been introduced as powerful, label-free alternatives to traditional bioassays (19–29). In this study, we conducted large-scale analysis under standard laboratory conditions using an automated UHT platform based on desorption electrospray ionization (DESI), integrating custom and commercial software, high–precision robotics, and analytical instrumentation (30). DESI operates via online microextraction-desorption as a charged solvent spray impacts individual spots on a high-density sample array prepared with minimal volumes (50 nL), allowing for direct subsecond analysis of complex biological samples (30–32). Additionally, we adapted this UHT approach for use with more widely accessible commercial instrumentation, enabling smaller-scale studies involving authentic CWAs in a specialized facility. We combined a simple fluid handling workstation for automatic sample preparation and decontamination with a conventional liquid chromatography (LC) MS instrument for HT direct injection (i.e., chromatography-free) electrospray ionization (ESI), providing an overall throughput of ca. 1 sample per minute. In both cases, the raw MS response was converted to reaction progression via automated data analysis using appropriate calibrations (*SI Appendix*, Figs. S1 and S6). This MS-based bioassay methodology was first thoroughly validated using both authentic and surrogate (i.e., nonhuman) cholinesterases (ChEs), for which complete kinetic characterizations were performed (Fig. 1*B* and *SI Appendix*, Figs. S2–S4 and Table S1) and found to be consistent with literature (33–40). These validation results align with reports on the significant differences among species ChEs (38, 41–47), reemphasizing the need for caution when extrapolating surrogate results to human systems.

Using these MS technologies, we characterized the inhibition of human acetylcholinesterase (*h*AChE) by three amidine A-series agents scheduled by the CWC: A-230, A-232, and A-234, as well as two phosphoramidate ester pesticides (methamidophos and fenamiphos; *see* structures in *SI Appendix*, Fig. S5), which share the A-series P-N bond. This is a quantitative experiment on AChE inhibition by the amidine family of A-series CWAs. Rapid and automated MS-based characterization of the *h*AChE inhibition by this set of toxic organophosphorus compounds was performed using both pre- (Fig. 1*C*; see also *SI Appendix*, Fig. S5 and S7) and coincubation (Fig. 1*D* and *SI Appendix*, Fig. S8) methodologies common to CWA adduction studies, with the latter being preferable for higher potency inhibitors (48–50). Overall, the results aligned with the expected kinetic models (42, 49, 51–53) and allowed for estimation of bimolecular inhibition rate constants (*k*_i_) under pseudo-first-order conditions (Table 1; see also *SI Appendix*, Table S3). Notably, the *k*_i_ values obtained were remarkably consistent between assay methodologies and confirmed that the A-series agent potencies are comparable to those of traditional CWAs like sarin and VX (ca. 4 × 10^7^ and 1 × 10^8^ M^−1^ min^−1^, respectively) (8), rather than significantly greater as other reports suggest (11, 54). Additionally, we found A-230 to be twice as potent as A-232 or A-234, consistent with the trend of phosphonates being generally more potent ChE inhibitors than phosphates (8, 49) and in agreement with theoretical calculations (16) as well as recent experimental studies using cell-based sensors (55). Note that the two phosphoramidate pesticides were found to be significantly weaker, as expected (49, 56).

**Table 1.** Bimolecular inhibition rate constants, *k_i_*, for amidine A-series CWAs and phosphoramidate pesticides*

Inhibitor	Incubation	*k*_i_ (M^−1^ min^−1^)
A-230	Pre	(8.6 ± 0.3) × 10^7^
	Co	(8.3 ± 0.2) × 10^7^
A-232	Pre	(4.6 ± 0.8) × 10^7^
	Co	(4.5 ± 0.1) × 10^7^
A-234	Pre	(4.8 ± 0.2) × 10^7^
	Co	(4.8 ± 0.1) × 10^7^
Methamidophos	Pre	(1.7 ± 0.1) × 10^3^
Fenamiphos	Pre	200 ± 3

^*^Determinations using live CWAs were carried out in a specialized facility via HT-ESI-MS at room temperature (20 °C). Pesticides were studied in a standard laboratory environment via UHT-DESI-MS under incubation at 37 °C.

As demonstrated, successful implementation of HT-MS using common analytical instrumentation allows experimentation at scale with live CWAs in specialized environments. However, due to the extensive chemical and experimental spaces that need exploration, efficient countermeasure candidate discovery requires a higher throughput typically available only at dedicated core facilities. To address this limitation, we developed a workflow for generating, purifying, and transferring CWA-adducted ChEs for remote countermeasure candidate discovery at UHT screening facilities unable to handle CWAs themselves (see *SI Appendix* for experimental details). This pipeline ensured minimal concentrations (sub-ng/mL) of free agents in the transferred materials, significantly below reported CWA LD_50_ values. Moreover, to enable this transfer, we characterized the aging process of the adducted ChEs (Fig. 1*A*), finding no significant aging of any of the A-series adducts after a month (*SI Appendix*, Fig. S9 and Table S4). These results verify the remarkable stability of the covalent A-series-ChE complexes, which has been suggested previously using selected agents and surrogate molecules (13, 54).

Applying the developed workflow, samples of *h*AChE adducted with the three A-series agents, as well as VX and *p*-nitrophenol ethyl methylphosphonate (NEMP, a well-established VX surrogate) (57, 58), were prepared, transferred, and used for countermeasure candidate screening via UHT-DESI-MS in a standard laboratory environment. This automated platform enabled accelerated screening of oxime candidates across various reactivation times, including necessary controls for their intrinsic inhibitory activity (3,500 samples in under 45 min), in the absence of common interferences (i.e., “oximolysis”) from traditional colorimetric reactivation studies (59–61) (*SI Appendix*, Fig. S10).

The library that was screened against VX- and NEMP-adducted *h*AChE was synthesized in-house (*SI Appendix*) and contained established oxime reactivators as well as experimental candidates designed for enhanced blood–brain barrier (BBB) permeability, including ten new imineoximes based on the monoisonitrosoacetone (MINA) scaffold (62–65) for which this is the first CWA reactivation report. The structures and identifying code numbers for all the oximes evaluated are shown *SI Appendix*, Table S5. Overall, well-known ionic oxime reactivators (66) (***29***–***32***) showed the highest efficacy, although many neutral oximes with better BBB permeability were also effective (Fig. 1*E*). Among the hydroxyiminoacetamide-based oximes (***1***–***14***) (64, 67–69), the basicity of the distal nitrogen was found crucial for reactivation potency. Those with tert-butyloxycarbonyl (BOC) protection (***7***–***9***, ***11***) or aromatic (***2***) nitrogens were generally inactive (cf. ***2*** and ***3*** or ***10*** and ***11***), whereas those with saturated *N*-heterocycles (***1***, ***3***, ***14***) were found to be most potent. Notably, LLNL-02 (***12***) demonstrated high reactivation potency despite its increased steric hindrance and lipophilicity (68). All MINA-based imineoximes showed comparable activity (***15***–***24***), whereas longer aliphatic chains in previously reported guanidine candidates (63) led to reduced potency (***25***–***28***). Across all candidate oxime classes, morpholine groups (***4*** and ***23***) led to lower reactivation efficiencies following the aforementioned basicity trend. Note that, on a per oxime basis, VX and NEMP-adducted AChE demonstrated comparable reactivation across the panel (Fig. 1*E* and *SI Appendix*, Table S6), reaffirming NEMP as an appropriate surrogate for VX-countermeasure development.

Unlike traditional CWAs, reactivation of A-series adducts by oximes has been frequently reported as unlikely, a factor accentuating their ascribed threat (2, 11). We tested this hypothesis using well-established oximes (***29***–***32***) known for their efficacy against conventional CWAs (8, 66). Contrary to previous reports (11, 54), we found that bispyridinium oximes (***30***–***32***) can indeed reactivate A-series-adducted *h*AChE in vitro over the course of four hours, though 2-PAM (2-pralidoxime, ***29***) showed no significant activity (Fig. 1*F*). These results were confirmed through dose–response experiments, identifying HI-6 (asoxime, ***30***) and MMB-4 (methoxime, ***32***) as the most effective candidates, with reactivation degrees following the trend A-230 > A-234 > A-232 (Fig. 1*G* and *SI Appendix*, Fig. S11). Longer time-course experiments (Fig. 1*H*; see also *SI Appendix*, Table S7) revealed HI-6 achieved over 60% reactivation of A-230-adducted *h*AChE in less than 6 h, being >threefold more effective than against A-234 and A-232. Surprisingly, after 72 h, 2-PAM did indeed exhibit reactivation, though modest (~25% for A-230), indicating some in vitro activity. However, this finding probably lacks countermeasure relevance due to the rapid in vivo metabolism and clearance of 2-PAM. Note that no spontaneous reactivation was observed on any of the evaluated time scales (up to 72 h), pointing again to the stability of the amidine A-series-ChE adducts.

The enhanced performance of bispyridinium oximes relative to 2-PAM likely results from their two cationic sites, which have been reported to enhance binding to AChE via interactions with both its peripheral anionic site as well as its active-site gorge (70, 71). This synergy is hypothesized to enable appropriate orientation for the nucleophilic attack that leads to the observed reactivation. However, it is worth noting that minor structural differences among the bispyridinum oximes, for instance, in the linker between the charged rings, can lead to significant changes in activity, even in an agent-dependent manner (72, 73). Thus, large-scale structural studies, which are now facilitated by the decentralized (U)HT workflow demonstrated here, are required to gain further insights into the observed activity against the A-series-AChE complexes, as well as to optimize the bispyridinium scaffold toward more medically relevant candidates. Note that discovering effective candidates also requires rapid, large-scale synthetic efforts to efficiently generate diverse oxime libraries and to fully leverage high-throughput biochemical screening. Coordinating these parallel campaigns of oxime synthesis and bioassessment remains a significant challenge that can be addressed by combining the previously demonstrated capabilities of UHT-DESI-MS for both organic reaction screening (74, 75) and small-scale synthesis (76, 77) with the label-free bioactivity assays described in this work.

Overall, this study characterized the largely unknown behavior of A-series CWAs in vitro using label-free MS-based approaches. Specifically, we demonstrate i) the inhibitory potency of A-series CWAs toward *h*AChE is comparable to traditional nerve agents; ii) A-series—*h*AChE adducts are stable, with no significant aging detected over a month postexposure and no spontaneous reactivation over more than 72 h; and iii) these CWAs are less susceptible to reactivation by traditional oxime therapies, although bispyridinium-based candidates can reactivate A-series-adducted *h*AChE with modest potency in in vitro experiments. This last finding challenges widely accepted conclusions based on anecdotal clinical data (2) and strengthens the case for broadly establishing HI-6 and MMB-4 as standard therapies for CWA exposure (41, 78), while also supporting further development and evaluation of novel bispyridinium-based oxime countermeasure candidates. Additionally, this work highlights the broad utility of (U)HT-MS for label-free biological activity assays and its potential to accelerate large-scale drug discovery campaigns. As shown, with full automation and minimal sample requirements, (U)HT-MS enables safe characterization of authentic CWAs in surrogate-free systems via extensive quantitative measurements. Moreover, the reported workflows represent a powerful capability to support decentralized translational science and showcase (U)HT-MS as an agile and robust technology for developing novel countermeasures candidates against emerging toxic threats.

## Materials and Methods

### Safety Considerations.

CWAs are acutely toxic compounds that can harm exposed individuals at extremely small doses. Only properly trained personnel in a certified laboratory possessing the adequate equipment to carry out their synthesis, subsequent purification, and use should handle these highly toxic CWAs. The Forensic Science Center (FSC) at Lawrence Livermore National Laboratory (LLNL) is a United States Designated Laboratory for the Organisation for the Prohibition of Chemical Weapons (OPCW), which oversees verification and compliance with the CWC. This accreditation authorizes the FSC to synthesize and handle small quantities of CWAs in support of OPCW analytical and verification activities. The handling and preparation of all CWA samples involves the use of proper protective personal equipment (viz., lab coat, safety glasses, butyl-based gloves with nitrile gloves underneath, and face shield) and is conducted inside an appropriate and certified chemical fume hood. All biochemical assays in this study utilized only dilute (de minimus) CWA solutions that were handled robotically in an adequately exhausted workstation. All labware in contact with CWA was appropriately decontaminated in bleach for at least two weeks. Further details on the workflows followed are provided below and in *SI Appendix*.

### Ultrahigh-Throughput Desorption Electrospray Ionization Mass Spectrometry (UHT-DESI-MS).

UHT biological assays which did not involve free A-series agents (i.e., only purified adducted enzyme or surrogate material) were carried out using two automated platforms based on DESI-MS (30). A third system (see below) was used for HT-ESI-MS studies with free agents in a specialized facility. Both UHT-DESI-MS systems combine fluid handling robotics and mass spectrometers equipped with DESI ion sources together with custom control software. The first-generation system, used for the characterization of the different cholinesterases (ChEs) as well as the phosphoramidate pesticides, was developed as part of the DARPA Make It program and it combines a Biomek i7 fluid handling workstation (Beckman Coulter, Brea, CA) and a LTQ XL linear ion trap mass spectrometer (Thermo Fisher Scientific, San Jose, CA) integrated via an articulated robot arm (SCARA PF3400, Precision Instruments, San Diego, CA). A 2D DESI system (Prosolia, Indianapolis, IN) allows UHT spot-to-spot analysis at a rate of 1 Hz. Overarching system control, including plate transfer, analysis, and initial data processing (i.e., RAW data parsing and targeted extraction) is achieved using a custom Python-based software termed CHRIS (79). The second-generation UHT-DESI-MS system used for candidate countermeasure screening and characterization was developed under the NCATS ASPIRE program in collaboration with Waters Corporation and Hamilton Robotics. This system combines a Vantage 2.0 (Hamilton Robotics, Reno, NV) fluid handling workstation with a Xevo^TM^ G2-XS quadrupole time-of-flight mass spectrometer equipped with a prototype AutoDESI ion source (Waters Corporation, Milford, MA). Hardware integration uses a custom enclosure, a track gripper, and an open landing position setup of the DESI source, whereas software integration proceeds via a custom seamless driver. DESI-MS analysis proceeds in a spot-to-spot manner with a throughput of up to 3 Hz. For both platforms, data analysis (i.e., ion ratio calculation, sample averaging, fitting) is carried out using a custom MATLAB application.

Independent of the platform used, the general workflow followed for all experiments includes three main steps (30). First, master 384-well microplates (polypropylene, V-bottom; Greiner Bio-One, Monroe, NC) are created following the specific assay conditions, typically by transferring the substrate and inhibitors, initiating the reaction by enzyme addition, and finally quenching the reactions by addition of ice-cold acetonitrile (two assay volumes). Incubation of all reactions explored via UHT-DESI-MS (i.e., enzyme characterization, pesticide studies, oxime screening and confirmation) was carried out offline at 37 °C. Second, 50-nL aliquots from each well on the microplates are automatically spotted onto custom-sized (4.5 × 3.3 × 0.074 in) soda lime glass-slides (Abrisa Technologies, Santa Paula, CA) coated with a Zytex G-115 porous polytetrafluoroethylene (PTFE) membrane (Saint-Gobain, Wayne, NJ) using a slotted floating pin-tool (V&P Scientific, San Diego, CA). The slides are coated in-house using a light film of low-VOC spray adhesive (Scotch Spray Mount, 3M, St. Paul, MN). All slides are prepared at least 24 h before use. Automation compatibility is achieved using standard microplate size 3D-printed holders in which the glass slides fit tightly. Up to 16 microplates can be spotted on a single PTFE-coated slide by offsetting the spotting position 1.125 mm between transfers, yielding high-density arrays of up to 6,144 samples per plate. The spotting density is user-defined and for most experiments was selected at 3,072 or 1,536 samples per plate with at least four replicates of each well plate being analyzed per run. For all experiments, dye marks are also pinned at predefined positions near the corners of the plate for the three-point positional calibration of the spotted array once delivered to the DESI stage. Third, the sample plates are automatically transferred to the DESI stage and quantitative UHT analysis proceeds directly from the spotted bioassay mixtures. A single RAW file is acquired per plate with file parsing (i.e., assignment of the spectral data corresponding to each sample spot) occurring in quasireal time. Parsed data (which averages all the scans acquired per sample spot, typically 5 to 7) are used for further quantitative analysis.

For both systems, instrumental DESI-MS conditions were initially optimized for the analytes of interest (i.e., choline, acetylcholine, butyrylcholine). Automated monitoring of reaction progress proceeded via calculation of conversion ratios based on the MS intensities of the species of interest and their calibration to absolute concentrations using an external calibration curve. Details on optimization and conversion ratio calibrations are described in *SI Appendix*.

### High-Throughput Electrospray Ionization Mass Spectrometry (HT-ESI-MS).

Following the concept of the UHT-DESI-MS platform, we combined a Lynx LM1200 robotic liquid handler equipped with a VVP96 pipetting head (Dynamic Devices, Wilmington, DE) and a Q Exactive™ HF-X Orbitrap™ MS coupled to a Vanquish Flex HPLC (Thermo Fisher Scientific, San Jose, CA) for the HT biological assays involving free A-series agents. These studies were carried out at LLNL. The fluid handling workstation was actively exhausted via a connection to the building venting system with adequate capacity for safe operation with up to 1 ppm concentrations of free, active chemical agent. Using this workstation, all samples were automatically prepared in MS-compatible deep 96-well plates (Thermo Fisher Scientific, Waltham, MA). Automatic routines were programed (Method Manager software V1.4.8054) in the handler to carry out the assays and to discard any tips that came in contact with agents into dedicated racks for decontamination with bleach. Note that all the assays were simultaneously quenched and diluted in a 1:1,000 proportion with acetonitrile. This reduced all final maximum agent concentrations down to 100 ppt before the plates were transferred out of the liquid handler and transferred for subsequent analysis, as well as facilitated ESI analysis of the diluted bioassay mixtures. All labware were thoroughly decontaminated with bleach for over two weeks after use before proper final disposal. All reactions explored via HT-ESI-MS (i.e., those involving free A-series agents) were incubated on the fluid handling workstation deck at room temperature (20 °C).

The analytical system was configured in a chromatography-free mode, with the autosampler of the liquid chromatograph being directly connected to the mass spectrometer via a heated ESI source, bypassing the use of a chromatographic column (i.e., direct injection). An isocratic elution profile (flow rate = 0.5 mL/min) of 0.8 min in length was implemented with a 1:1 mixture of 0.1% formic acid in water and 0.1% formic acid in acetonitrile (0.2 min), followed by a 100% aqueous wash (0.4 min), and a final wash/re-equilibrate step at the initial 1:1 mobile phase composition (0.2 min). This allowed for an overall throughput of ~1 min per sample (accounting for sample injection time) and an integrated line and ion source wash to prevent sample carryover and potential clogging of the lines by the already heavily diluted biological matrix. Automated data analysis proceeded via a custom MATLAB script following the same workflow of calculation and calibration of MS conversion ratios as carried out for the UHT-DESI-MS analysis. Further details on instrumental conditions, absolute quantitation, and data processing are provided in *SI Appendix*.

### Method Validation Through Label-Free Characterization of Cholinesterases.

Four commercially available cholinesterases were characterized via UHT-DESI-MS: recombinant human acetylcholinesterase expressed in HEK 293 cells (*h*AChE), type V-S electric eel acetylcholinesterase (*ee*AChE), butyrylcholinesterase from equine serum (*eq*BChE), and butyrylcholinesterase from human serum (*h*BChE). The native AChE substrate, acetylcholine, was used for characterization of both AChEs, whereas butyrylcholine was utilized for both BChEs. A total of 15,360 samples were analyzed for the kinetic characterization of each enzyme (16 replicates of 24 time-point curves for each one of the 40 concentrations explored). Further studies were conducted to assess the substrate cross-reactivity (i.e., activity of AChEs with butyrylcholine and BChEs with acetylcholine), as well as noncovalent inhibition using reported selective AChE and BChE inhibitors ((-)-huperzine A and ethopropazine, respectively). More than 500 measurements were carried out for each experimental condition in these experiments. The results obtained through this entire validation are described in detail in *SI Appendix* and largely agree with literature reports.

### Label-Free Characterization of Acetylcholinesterase Inhibition by Organophosphorus Compounds.

Two organophosphorus pesticides (methamidophos and fenamiphos) and three A-series agents (A-230, A-232, and A-234) were characterized in an automated fashion using the (U)HT-MS methodologies described above. Pesticides were characterized via UHT-DESI-MS at 37 °C in a standard BSL2 laboratory space, whereas authentic A-series agents were studied via HT-DESI-MS at room temperature (20 °C) in a specialized facility. In all cases, preincubation assays were carried out by first mixing the inhibitor (at several concentrations) and enzyme. Then, after a variable time, the substrate (acetylcholine) is added without significant dilution for a fast reaction (i.e., significantly shorter than the preincubation time) within the linear regime of the enzyme-mediated substrate hydrolysis. This hydrolysis reaction time was fixed in all experiments via quenching with ice-cold acetonitrile. Comparison of initial reaction rates of the inhibitor-exposed samples to uninhibited controls across different inhibitor concentrations allowed for the determination of observed reaction rates (*k_obs_*) using the ratio of the initial reaction velocities for the inhibited samples (*v_i_*) and the uninhibited controls (*v_0_*). The *k_obs_* values are then used for the estimation of bimolecular inhibition rate constants (*k_i_*) of the AChE-pesticide system under pseudo-first-order conditions (48, 49, 80). For the A-series agents, an additional independent characterization was carried out using a coincubation methodology that allows for larger agent concentrations to be explored. In this case, acetylcholine and the A-series agents were initially mixed before the addition of the enzyme and the reaction progress was monitored over the linear regime of the uninhibited enzymatic reaction. The *k_obs_* calculated at each inhibitor concentration via nonlinear regression (time vs. assay response, using the initial rate of the uninhibited enzyme as a fixed parameter) were then utilized to estimate the bimolecular *k_i_* values via linear regression (42, 51–53). Further experimental details as well as associated equations and extended results are included in *SI Appendix*.

### Generation, Purification, and Evaluation of Adducted Cholinesterases for Multifacility Studies.

All our multifacility studies focused only on *h*AChE. For adduction and purification, *h*AChE was suspended in 100 mM potassium phosphate pH 8 to a final concentration of 0.2 mg/mL. Samples were spiked with in-house made VX, NEMP, A230, A232, or A234 (each at 100 µg/mL in methanol) at a 10-fold molar excess. A negative control sample was spiked with only methanol. A spiked *h*AChE solution was transferred to a passivated Amicon® Ultra 3 kDa molecular weight cutoff (MWCO) spin filter (Millipore, Burlington, MA) and washed three times with 100 mM potassium phosphate pH 8 to remove unreacted agent. Washed protein was recovered from the filter with a minimal volume of 100 mM potassium phosphate pH 8 and protein quantitation was performed using the Qubit™ Protein Assay kit on a Qubit™ 4 Fluorometer (Invitrogen, Waltham, MA). Samples were diluted to a final concentration of 1 µg/mL in 0.1% BSA (Sigma Aldrich, St. Louis, MO) solution in 100 mM potassium phosphate pH 8.0. The free concentration of each agent after purification was determined using a standardized quantitative LC–MS/MS method (see details in *SI Appendix*). Using this methodology, we validated that the level of free agent in any of the samples shipped for UHT reactivation screening studies was safe for handling in a standard laboratory facility. In all cases, the concentration of agent achieved was sub ng/mL, significantly below reported median lethal dose (LD_50_) values for CWAs.

### Evaluation of Aging of A-Series Adducted Cholinesterases.

These studies used *eq*BChE (Sigma Aldrich, St. Louis, MO) as model. The enzyme was suspended in 100 mM potassium phosphate pH 6 to a final concentration of 0.2 mg/mL, aliquoted into Protein LoBind® Tubes (Eppendorf, Framingham, MA) and was stored at 4 °C for spiking over the study duration. The pH of the buffer was adjusted to pH 6, in contrast to pH 8, in order to accelerate the aging rate to encourage a “worst-case scenario” based on previously published research (38). Samples were retrieved at the desired time points, spiked with agent, and incubated at 37 °C until the end-date of the study. Samples were spiked with A-230, A-232, or A-234 (100 µg/mL in methanol) at four-times molar excess; a control sample was spiked with methanol. Sample spiking was staggered such that the following durations were generated: 1 mo, 2 wk, 1 wk, 16 h, and 0 h. All samples were processed in a single batch to minimize variability due to sample preparation. Digestion of 75 µL *eq*BChE was performed by adding 40 µL 100 mM potassium phosphate pH 6 and 10 µL of 2 mg/mL pepsin (Sigma Aldrich, St. Louis, MO) in 5% formic acid and incubating at 37 °C for 30 min. Digestion was stopped and undigested material was removed by transferring the solution to an Amicon® Ultra 10 kDa MWCO spin filter (Millipore, Burlington, MA). MWCO filter flow-though was transferred to an autosampler vial with insert (Agilent, Santa Clara, CA) for analysis. Peptide analysis was carried out via LC–MS/MS as described in *SI Appendix*.

### Synthesis and Characterization of Oxime Candidates.

The panel of oximes screened was synthesized in-house as described in *SI Appendix*. The structures and code numbers utilized to identify the different oximes are included in *SI Appendix*, Table S5. To our knowledge, this is the first report of the imineoxime candidates ***15*** through ***24*** being evaluated as reactivators for CWA exposure.

### Ultrahigh-Throughput Screening of Oxime Candidates as Reactivators.

Oxime candidate screening was carried out similarly to all other UHT-DESI-MS previously described. CWA-adducted enzyme (*h*AChE) as well as nonadducted controls (subjected to the same purification process) were shipped from LLNL, received at Purdue overnight, and used immediately. Panel screening experiments were carried out for VX- and NEMP-adducted *h*AChE using 100 µM final oxime concentration and three reactivation time points: 30 min, 1 h, and 4 h. Dose–response confirmation experiments for selected oximes were conducted with a fixed 4-h reactivation time, varying the oxime concentration across six orders of magnitude (1 nM to 1 mM). For the A-series-adducted *h*AChE, established oximes (***29***–***32***) were evaluated under the same conditions used for the VX and NEMP cases. Lengthier reactivation attempts (up to 72 h) were conducted with the most promising oxime, HI-6 (***30***), as well as the seemingly unactive, but well-established 2-PAM (***29***). Further experimental details are described in *SI Appendix*.

## Supplementary Material

Appendix 01 (PDF)

## Data Availability

All study data are included in the article and/or *SI Appendix*.
