# Supplementary material for: Accelerating countermeasure candidate discovery for A-series chemical warfare agent exposure
Source: Proc Natl Acad Sci U S A. 2025 Jul 17;122(29):e2512471122. doi: 10.1073/pnas.2512471122 (PMC12304963; doi:10.1073/pnas.2512471122)
Supplement: Supplementary file 1 — Appendix 01 (PDF) [file pnas.2512471122.sapp.pdf]

Supporting Information for
Accelerating Countermeasure Candidate Discovery for A-Series Chemical Warfare Agent Exposure

Nicolás M. Morato,[a] Katelyn E. Mason,[b] Todd H. Corzett,[b] Carlos A. Valdez,[b] Teneile M. Alfaro,[b] Saphon Hok,[b] R. Graham Cooks,[a,c]* and Brian P. Mayer[b]*

[a] Purdue Institute for Cancer Research, Purdue University, West Lafayette, IN 47907, USA.
[b] Forensic Science Center, Lawrence Livermore National Laboratory, Livermore, CA 94550, USA.
[c] Department of Chemistry, Purdue University, West Lafayette, IN 47907, USA.

*Corresponding authors: Brian P. Mayer and R. Graham Cooks
Email: mayer22@llnl.gov and cooks@purdue.edu

This PDF file includes:

Supporting text
Figures S1 to S11
Tables S1 to S7
Equations S1 to S11
SI References

Supporting text

Ultrahigh-throughput desorption electrospray ionization mass spectrometry (UHT-DESI-MS)

Experimental conditions. For the first-generation system (1, 2), DESI solvent spray flow was set to 3 µL/min with nitrogen (150 psi) as nebulizing gas and a spray voltage of 5 kV. The capillary temperature was adjusted to 300 °C, the tube lens voltage to 20 V, and the capillary voltage to 46 V. Mass filtering was carried out by selecting a wide isolation window for selection of all analytes in each experiment (enzymatic substrate and product) with no collision energy applied and a short activation time (5 ms). Automatic-gain control was utilized in all experiments, with the maximum scan time set to 150 ms, allowing for ~4 scans to be acquired per spot and achieving a throughput of ~1 second per sample. For the second-generation platform lower DESI solvent flow (2 µL per min), nebulizing gas pressure (15 psi; regulated on the stage), and spray voltages (0.6 kV) were used. A home-built heated transfer capillary was set to 450 °C, whereas the ion source temperature was 125 °C and the sampling cone voltage was 40 V. The total scan time was fixed to 100 ms (86 ms of scan and 14 ms of interscan time) with an overall throughput of ~900 ms per sample (these settings allowed for the acquisition of 7 scans per spot). Low-mass ion transmission was improved by optimizing the instrument RF settings. The final selected conditions kept the default StepWave and ion guide RF amplitude values at 300 and 350 V, respectively, with a lower collision cell RF offset of 300 V (cf. default at 600 V). A manual quadrupole profile was also selected with a 100% ramp time between 50 and 200 *m/z* units. Target enhancement (i.e., synchronization of the time-of-flight conditions) was also utilized as discussed in the next section. In all cases analysis was carried out in the positive ion mode and methanol was used as DESI spray solvent. Note that for all UHT-DESI-MS experiments only LC-MS grade solvents were utilized (CHROMASOLV Honeywell Riedel-de Haën, Charlotte, NC).

Absolute quantitation of cholinesterase reaction progress. Reaction progress was monitored by calculating a conversion ratio using the ion intensities of the substrate and product of the reaction as shown in **Equation S1**. These raw reaction conversions are then calibrated to absolute concentrations of choline produced through the reaction using an external calibration curve that correlates MS conversion ratios to absolute concentration ratios (3). Note concentration ratios follow an equation identical to that for the MS response but use absolute concentration values instead of ion intensities. Ideally, if there are no ionization efficiency differences between the reaction product and substrate, a unit slope and a negligible intercept are expected from this calibration. Deviations from this scenario indicate differences in ionization efficiency or in ion transmission and detection efficiency under the selected experimental conditions.

$$Conversion\ Ratio = \frac{I_{Product}}{I_{Product} + I_{Substrate}} \qquad \textbf{(Equation S1)}$$

Using the first-generation system (linear ion trap mass spectrometer) several calibration curves were run over multiple months, completing 384 independent replicates per calibration standard (i.e., concentration ratio), reaching 1,536 technical replicates per point for a single calibration (i.e., single overall concentration). A total of 40 overall concentrations (i.e., total amount of substrate and product) were explored to cover the range utilized for the Michaelis-Menten characterization of the cholinesterases (50 µM to 50 mM), representing 15,360 samples analyzed through the course of the calibration experiments for each substrate-product pair. The average calibration obtained for the choline-acetylcholine pair (*m/z* 104 and *m/z* 146, respectively) is shown in **Fig. S1A**. Residual analysis (**Fig. S1B**) indicated a second-order polynomial calibration would be more appropriate, suggesting likely differences in ion transmission and detection or ion suppression effects as the relative substrate-product ratio changes. Adjusted R^2 increased from 0.992 to 0.999 with the use of a second-order polynomial model, and the residual sum of squares (RSS) decreased an order of magnitude from 0.015 to 0.0014 by including a non-linear component. As expected, a stronger non-linear effect was observed in the more distant choline-butyrylcholine (*m/z* 174) pair (**Fig. S1C**).

In both cases no further modification of the instrumental conditions was carried out, and the calculated second-order calibration equations were used for estimation of reaction progression in all experiments. The robustness of the calibration curve obtained (coefficients of variance, CVs, under 15% in all cases) stems from the ability to quickly prepare and analyze many standards across the calibration range, and it showcases the capabilities of DESI-MS for rapid quantitative analysis. Note this capability was key to identifying the non-linear components on the instrumental response, which would be easy to miss if fewer calibration points were acquired across the curve range and probably disregarded to prevent overfitting if an overall lower number of samples had been analyzed.

Using the second-generation system, more extensive optimization of the mass spectrometer conditions was required to improve the low mass (especially under *m/z* 150) ion transmission. Without this optimization we observed that detection was biased towards the larger *m/*z substrates and sensitivity was compromised. To prevent this, we modified the default quadrupole profile and RF settings of the instrument. Decreasing any of the adjustable RF parameters (StepWave and ion guide amplitudes, or collision cell offset) led to preference for low mass ions, although reaching too low values (<150 V) compromised significantly the overall ion current. After combinatorial iterations, we identified as key parameter the RF collision cell offset, which we set to 300 V for sensitive detection of species in the range *m/z* 80-180. The target enhancement feature was also optimized, as we noticed that the *m/z* selected for enhancement has a significant effect on the preference for lower or higher *m/z* ions in this range, and thus on the shape of the calibration curves obtained, as discussed above. As observed in **Fig. S1D**, selection of values higher than *m/z* 107 led to mostly convex curves (suggesting the favoring of the higher mass substrate) whereas lower *m/z* values led to mostly concave ones (indicating a shift in the preference to the lower mass product). As values approach *m/z* 107, the curvature of the calibration lines decreases, indicating no preferential detection of either analyte, thus this value was selected for target enhancement and a linear calibration was used for absolute quantitation using this platform. For each calibration curve, 22 relative concentrations were prepared, with 32 independent replicates per sample. Four technical replicates were analyzed for each sample, yielding 3,072 calibration data points per curve.

Chemicals. For all experiments, choline chloride (99%; Acros Organics, Fair Lawn, NJ), acetylcholine chloride (99%; Acros Organics, Fair Lawn, NJ), and butyrylcholine chloride (≥98%; Sigma Aldrich, St. Louis, MO) stock solutions were freshly prepared in phosphate buffer (0.1 M, pH 8; Sigma Aldrich, St. Louis, MO) supplied with 0.1% bovine serum albumin (BSA; Sigma Aldrich, St. Louis, MO). These are the same reagents and matrix used in all the UHT bioassays. Note in further tests using human enzymes we attempted the use of 0.1% commercial human serum albumin (HSA) instead of BSA, but observed significant hydrolysis of the substrates in the control experiments (i.e., no cholinesterase) carried out in the presence of HSA, thus all the reported results utilized only BSA as protein stabilizer and to prevent non-specific protein binding (i.e., enzyme loss) to the utilized labware.

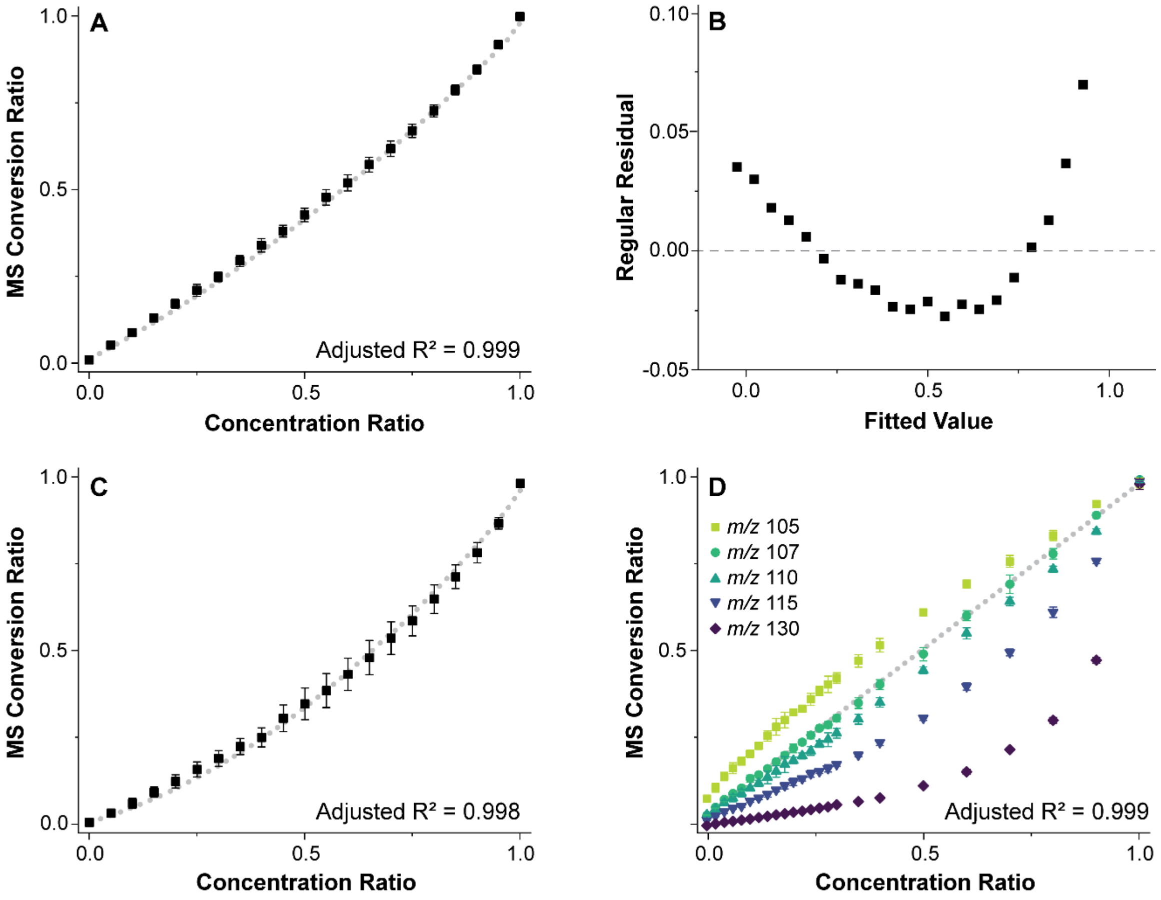

Fig. S1. Calibration of MS conversion ratios to provide reaction progress in terms of absolute concentrations. For the first-generation system, calibrations for both substrate-product pairs were recorded, with more than 15,000 samples analyzed per curve. The curve for acetylcholine-choline is shown in **A**, with its corresponding residual analysis in **B** indicating non-linear components. The curve for butyrylcholine-choline is shown in **C**. For the second-generation system, multiple calibration curves were obtained as a function of the target enhancement selection as shown in **D**. Each curve includes more than 3,000 data points. The selected enhancement value was *m/z* 107, which led to a linear calibration as shown. Error bars indicate standard deviations in all cases.

Label-free characterization of cholinesterases

Experimental details. Recombinant human acetylcholinesterase expressed in HEK 293 cells (*h*AChE), type V-S electric eel acetylcholinesterase (*ee*AChE), butyrylcholinesterase from equine serum (*eq*BChE), and butyrylcholinesterase from human serum (*h*BChE) were all procured from Sigma Aldrich (St. Louis, MO) and were used at a concentration of 10 ng/mL. Stock solutions of all enzymes were prepared, quantified (using an Invitrogen Qubit 4 Fluorometer and a Qubit Protein Assay Kit; Thermo Fisher, Waltham, MA), aliquoted and frozen until use. For all the reactions used for kinetic characterization, substrate and enzyme solutions were prepared at 2X concentrations and equilibrated at 37 °C. Reactions were automatically started by mixing equal volumes (10 µL) of enzyme and substrate solutions and later quenched at appropriate time points by addition 40 µL of ice-cold acetonitrile. Quenched reaction mixtures were immediately spotted and analyzed via UHT-DESI-MS. Kinetic characterization was carried out over a wide range of substrate concentrations (50 µM to 50 mM; 40 concentrations). The native AChE substrate, acetylcholine, was used for characterization of both AChEs whereas butyrylcholine was utilized for both BChEs. A total of 15,360 samples were analyzed for each enzyme (16 replicates of 24 time-point curves for each one of the 40 concentrations explored).

Kinetic characterization. The results of this detailed characterization are shown in **Fig. S2**. In the literature, significant substrate inhibition has been reported for AChE (4, 5), as we observed here, whereas substrate activation has been suggested for BChE (6). Two models were then used for the kinetic parameter estimation of each enzyme class: (i) Haldane and Webb-Radic for AChEs, and (ii) Michaelis-Menten and Webb-Radic for BChEs. The use of these models for the characterization of cholinesterases is common in the literature (7), with the Haldane equation being used to describe substrate inhibition (**Equation S2**), the Michaelis-Menten equation to describe standard steady-state kinetics (**Equation S3**), and the Webb-Radic model to captures the effect of charged substrate excesses (**Equation S4**) (8). The latter equation conveniently consolidates the known behavior of both AChEs and BChEs, which follow simple Michaelian kinetics with either neutral esters or low concentrations of charged substrates (e.g. acetylcholine, butyrylcholine) but diverge in behavior under high levels of such charged esters. Under these high concentration conditions, a second charged substrate molecule binds transiently at the peripheral anionic site (PAS) of the enzyme, located in the vicinity of the active site gorge. Occupation of the PAS then modifies the activity of the enzyme via steric effects and/or conformational changes, allosterically affecting its catalytic efficiency (7, 8). This phenomenon is experimentally observed as strong substrate inhibition in the case of AChEs and more subtle substrate-induced activation in BChEs.

$$v_0 = \left(\frac{v_{max}}{1+K_M/[S]+[S]/K_{SS}}\right) \qquad \textbf{(Equation S2)}$$

$$v_0 = \left(\frac{v_{max}}{1+K_M/[S]}\right) \qquad \textbf{(Equation S3)}$$

$$v_0 = \left(\frac{v_{max}}{1+K_M/[S]}\right)\left(\frac{1+b[S]/K_{SS}}{1+[S]/K_{SS}}\right) \qquad \textbf{(Equation S4)}$$

The results obtained from all models, which overall agree with previous studies (especially those using native ester substrates) (9–16), are compiled in **Table S1**. Almost identical results were obtained with both models for the AChEs, all cases indicating a slightly higher substrate affinity for *ee*AChE than its human counterpart (reflected by its ~1.5X lower K_m value), but a much higher maximum catalytic rate (v_{max}) and catalytic efficiency (k_{cat}/K_m) for the human enzyme compared to the electric eel one (~4 and ~2X, respectively). Additionally, *ee*AChE showed larger (~3X) substrate inhibition – also called dissociation – constant (K_{ss}) compared to *h*AChE, in agreement with its higher substrate affinity. In the case of the BChEs, large differences in K_m (~8X lower for *h*BChE compared to *eq*BChE), v_{max} (~2X higher for *eq*BChE compared to *h*BChE), and k_{cat}/K_m (~5X higher for *h*BChE compared to *eq*BChE) were observed between the human and equine enzymes. These conclusions are independent of the model utilized, as the *b* parameter estimated with the Webb-Radic model is identical for both enzymes.

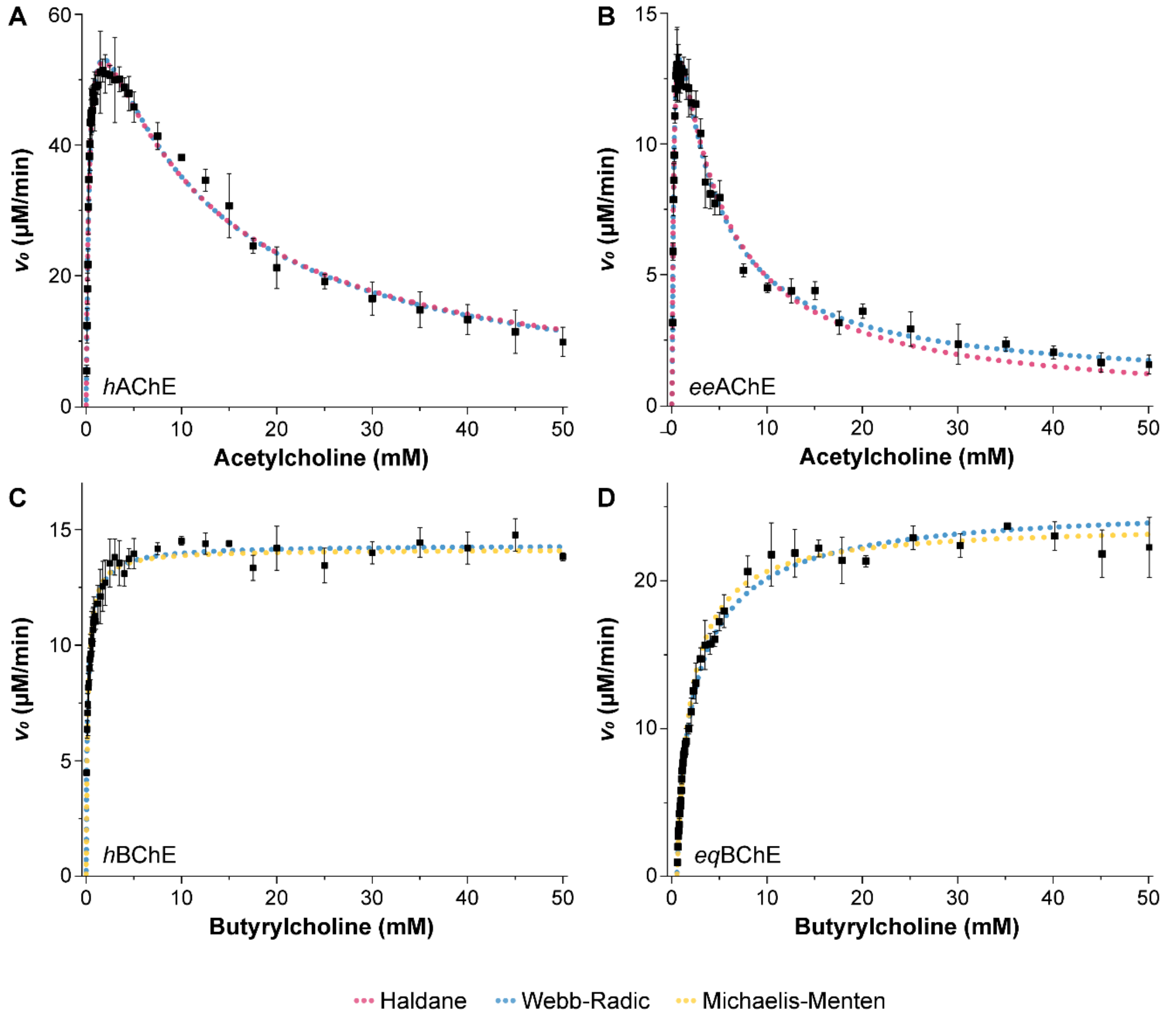

Fig. S2. Kinetic characterization across substrate concentrations for all the cholinesterases studied: *h*AChE (**A**), *ee*AChE (**B**), *h*BChE (**C**), and *eq*BChE (**D**). More than 15,000 samples per analyzed for each enzyme across 40 concentrations over a three-order of magnitude range (50 µM – 50 mM). For each enzyme the fitting results with different kinetic models are included. For AChEs the Haldane (H) and Webb-Radic (WR) models were utilized, whereas for BChEs the standard Michaelis-Menten (MM) and the WR models were considered. In general, similar adjusted R^2 values were obtained for *h*AChE (H 0.981 vs. WR 0.978) and *ee*AChE (H 0.988 vs. WR 0.983), although for *h*BChE (MM 0.953 vs. WR 0.984) and *eq*BChE (MM 0.987 vs. WR 0.998) the WR model seems to provide a better fit, likely due to the consideration of substrate activation. In all cases error bars indicate standard deviations across 16 replicate experiments for initial rate determination (24 time points each for monitoring reaction progression) at each one of the concentrations evaluated.

Note for both AChEs and BChEs, as mentioned above, the Webb-Radic model has been proposed as an adequate description of their kinetics with charged substrates, with the *b* parameter indicating substrate inhibition ($b < 1$) or activation ($b > 1$) due to allosteric effects from the excess of charged substrates (which at low concentrations reduce to traditional Michaelis-Menten kinetics, $b = 1$) (8). No significant differences in the goodness-of-fit measures were observed across models for the AChE data whereas better performance was obtained for the Webb-Radic model for the BChEs, suggesting substrate activation, as expected. In all cases fitting was carried out using OriginPro 2023b. It is worth highlighting that the UHT of the platform provides a unique capability for rapidly

studying in a very detailed fashion (tens of concentrations over a range orders of magnitude wide) the kinetics of an enzymatic system thus capturing all the above-discussed subtleties. Note that the significant cross-species differences observed (including also those regarding reversible inhibition as described below) align with the literature (14, 15, 17–19) further showcasing the utility of the method.

Table S1. Kinetic parameters estimated for all the cholinesterases evaluated under identical experimental conditions (phosphate buffer 0.1 M pH 8 with 0.1% BSA; 37 °C) using acetylcholine and butyrylcholine as substrates for AChEs and BChEs, respectively. For each enzyme the fitting results with different kinetic models are included: Haldane (H), Webb-Radic (WR) and Michaelis-Menten (MM). In all cases the estimated standard errors for the fitting parameters are indicated. N.S. denotes that the parameter is not significantly different than zero.

**Enzyme**	**Model**	**K_m (μM)**	**v_{max} (μM min^{-1})**	**K_{ss} (M)**	**b**	**k_{cat} (x10^5 min^{-1})**	**k_{cat}/K_m (x10^9 M^{-1} min^{-1})**
*h*AChE	H	355 ± 28	73 ± 3	9.6 ± 0.7	-	4.7 ± 0.2	13 ± 1
	WR	368 ± 32	76 ± 3	9.2 ± 0.8	N.S.	4.9 ± 0.2	13 ± 1
*ee*AChE	H	212 ± 22	19.8 ± 0.8	3.3 ± 0.3	-	1.29 ± 0.05	6.1 ± 0.7
	WR	282 ± 37	24 ± 2	2.2 ± 0.3	0.04 ± 0.01	1.6 ± 0.1	5.5 ± 0.9
*h*BChE	MM	194 ± 11	14.2 ± 0.1	-	-	0.923 ± 0.006	4.7 ± 0.3
	WR	60 ± 6	7 ± 3	0.4 ± 0.1	2.2 ± 0.8	0.4 ± 0.1	8 ± 2
*eq*BChE	MM	1466 ± 66	23.8 ± 0.3	-	-	1.55 ± 0.02	1.06 ± 0.02
	WR	550 ± 55	12 ± 2	3.8 ± 1.1	2.1 ± 0.3	0.8 ± 0.1	1.4 ± 0.3

Substrate cross-reactivity. Further characterization of the group of enzymes was carried out through substrate cross-reactivity experiments using 1 mM of acetylcholine or butyrylcholine and 10 ng/mL of enzyme. Samples were prepared identically as in the kinetic monitoring. Reaction progress (**Fig. S3**) and initial rates for one substrate across the enzymes were compared. Note appropriate control experiments, in particular, reactions without the presence of enzyme were run (this was also done for the kinetic monitoring), and no significant spontaneous hydrolysis of the ester substrates was observed over the time monitored, this is another advantage of the label-free methodology. Eight independent reactions were carried out, with each time point being analyzed in quadruplicate, for a total of 32 measurements per time point and >700 samples per curve.

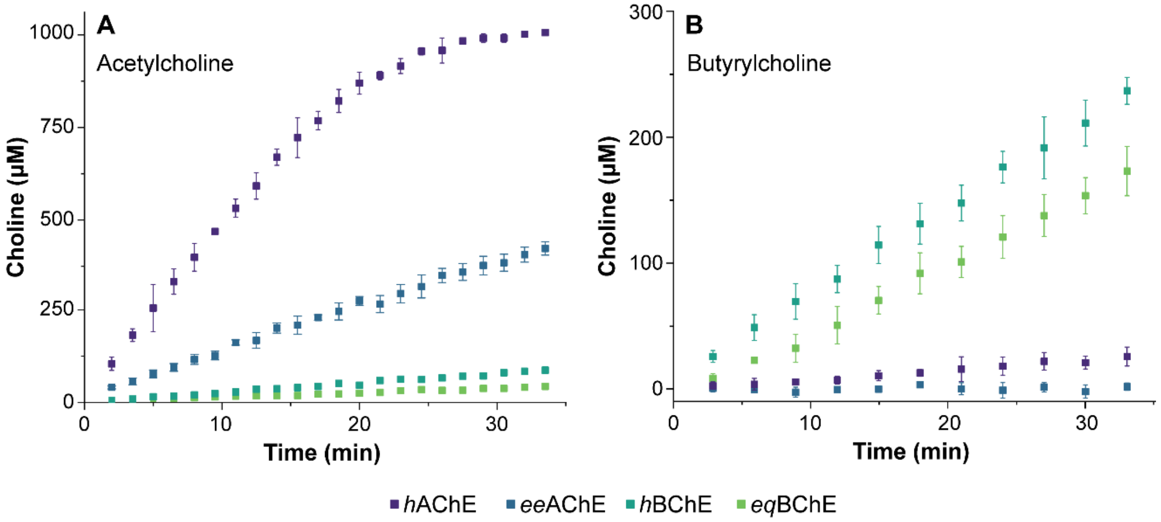

Fig. S3. Cross-reactivity experiments using acetylcholine (**A**) and butyrylcholine (**B**) as substrates for all the enzymes. The results show progress curves with error bars representing standard deviations (n = 32). In this experiment, the estimated initial reaction rates for acetylcholine were: *h*AChE = 45.6 ± 0.5 μM/min, *ee*AChE = 12.1 ± 0.2 μM/min, *h*BChE = 2.57 ± 0.05 μM/min, and *eq*BChE = 1.14 ± 0.03 μM/min, whereas for butyrylcholine were *h*AChE = 1.19 ± 0.05 μM/min, *ee*AChE = 0.05 ± 0.08 μM/min (not significantly different than zero), *h*BChE = 8.3 ± 0.2 μM/min, and *eq*BChE = 10.4 ± 0.1 μM/min.

Reversible inhibition. Finally, we characterized the non-covalent inhibition of all cholinesterases by (-)-huperzine A and ethopropazine (Cayman Chemical, Ann Arbor, MI), reported as selective inhibitors of AChEs and BChEs, respectively (20, 21). In this case, inhibitor, substrate and enzyme solutions were prepared in 3X concentrations (final substrate and enzyme concentrations of 1 mM and 10 ng/mL, respectively), and mixed in that order, with the enzyme addition starting the reaction. Final assay volumes of 15 µL were obtained and simultaneously quenched with 30 µL of ice-cold acetonitrile for end-point determinations of activity. Quenching time was selected to be within the linear regime of the reaction progress curve for each pair of enzymes (5 min for *h*AChE, 15 min for *ee*AChE, 30 min for both *eq*BChE and *h*BChE and *eq*BChE). Clear differences across enzyme classes and species were observed, as shown in **Fig. S4**, and in agreement with the literature (16, 22–24). Four independent reactions were carried out, with each inhibitor concentration being analyzed in quadruple, for a total of 16 data points per inhibitor concentration and >500 measurements per curve. The concentration ranges explored covered 9 orders of magnitude (pM to mM). All dose-response curves were fitted to sigmoidal models and half-maximal inhibitory concentrations (IC_{50}) were estimated when appropriate using Origin Pro 2023b. In cases where no or too little activity was observed no fitting or IC_{50} estimation was carried out.

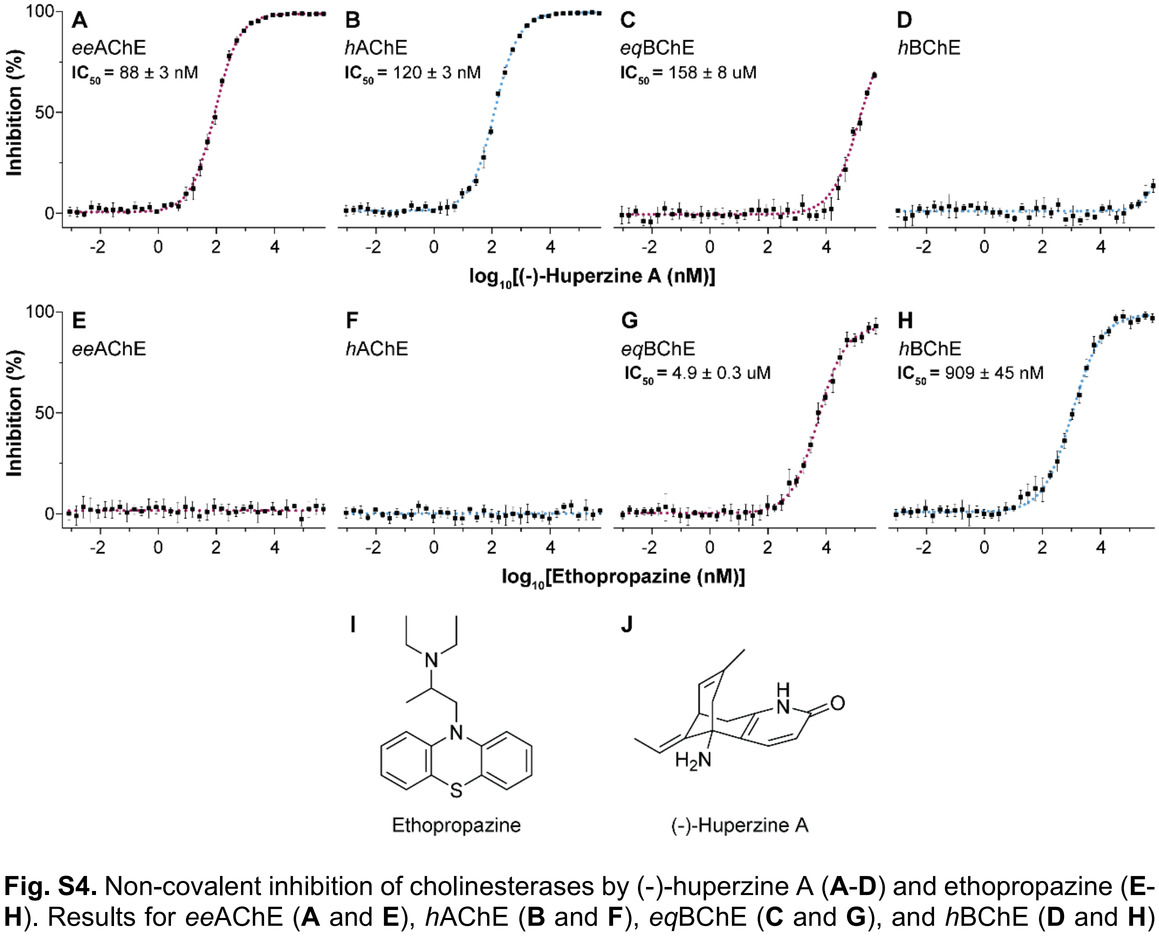

Fig. S4. Non-covalent inhibition of cholinesterases by (-)-huperzine A (**A**-**D**) and ethopropazine (**E**-**H**). Results for *ee*AChE (**A** and **E**), *h*AChE (**B** and **F**), *eq*BChE (**C** and **G**), and *h*BChE (**D** and **H**) are all included, as well as the structures of the two non-covalent inhibitors (**I** and **J**). In all cases error bars indicate standard deviations (n = 16). For the cases in which significant inhibitory activity was observed, sigmoidal fits together with IC_{50} values and their estimated standard errors are included.

Characterization of acetylcholinesterase inhibition by phosphoramidate pesticides

Experimental details. Initial experiments indicated the pesticides were weak inhibitors of AChE, thus their characterization was carried out using a pre-incubation methodology with no dilution (25). For this, enzyme and inhibitor were prepared at a 2X concentration, whereas substrate was prepared at a 10X concentration. The final concentrations of enzyme and substrate were 10 ng/mL and 1 mM, respectively, whereas the concentrations of pesticide were changed to assess dose-response effects. Pesticide stock solutions were prepared from commercial reagents as received (PESTANAL® analytical standards; Sigma Aldrich, St. Louis, MO) in DMSO and diluted so that the final DMSO concentration in the assay was less than 1%. Appropriate controls in the absence of pesticide and in the absence of enzyme were run on each plate. The covalent inhibition reaction (i.e., pre-incubation step) was started by automatic 1:1 mixing of enzyme and inhibitor solutions yielding a 20 µL assay in phosphate buffer 0.1 M pH 8 with 0.1% BSA. The prepared mixtures were incubated at 37 °C. At different time points over the course of three hours, 2 µL of substrate were added to each reaction mixture to start the AChE reaction and assess the amount of enzyme inhibited through the course of the pre-incubation. Finally, the acetylcholine hydrolysis was quenched after 3 min by the addition of ice-cold acetonitrile, and the samples were immediately analyzed via UHT-DESI-MS. This time is short compared to the pre-incubation time and is within the linear regime of the AChE-acetylcholine reaction. Complete dose-response curves at each of the 11 pre-incubation time points were obtained using this approach (**Figure S5A-B**).

Data analysis. Monitoring of a single pesticide concentration across pre-incubation times (**Figure S5C**) and comparison of the initial reaction rates of the pesticide-exposed samples to the pesticide-free controls (**Figure S5D**) allowed for the determination of observed reaction rates (k_{obs}) using the ratio of the initial reaction velocities for the inhibited samples (v_i) and the uninhibited controls (v_0). The k_{obs} values are then used for the estimation of bimolecular inhibition rate constants (k_i) of the AChE-pesticide system under pseudo-first-order conditions (**Figure S6D**). Following this assumption, only the highest pesticide concentrations were used for model fitting. The described k_i estimation follows **Equation S5** and **S6** as indicated in the literature (25–27). The results obtained for both *h*AChE and *ee*AChE are summarized in **Table S2** and the human enzyme data is also included in **Table 1** within the manuscript. Methamidophos showed equal potency for both AChEs, while fenamiphos had a five-fold lower k_i against *ee*AChE, likely due to different enzyme interactions with the larger compound. Notably, *h*AChE values aligned with previously reported rate constants from Ellman's assay (27–29), further validating this label-free MS methodology.

$$\ln(v_0/v_i) = k_{obs}t \qquad \textbf{(Equation S5)}$$

$$k_{obs} = [OP]k_i \qquad \textbf{(Equation S6)}$$

Table S2. Estimated constants for the inhibition reaction of *h*AChE and *ee*AChE by methamidophos and fenamiphos using the pre-incubation method. In all cases the standard errors from minimum least square estimation of the parameters are shown.

Inhibitor	Enzyme	ki (M^{-1} min^{-1})
Methamidophos	*h*AChE	(1.7 ± 0.1) × 10^3
	*ee*AChE	(1.6 ± 0.1) × 10^3
Fenamiphos	*h*AChE	200 ± 3
	*ee*AChE	30.7 ± 0.5

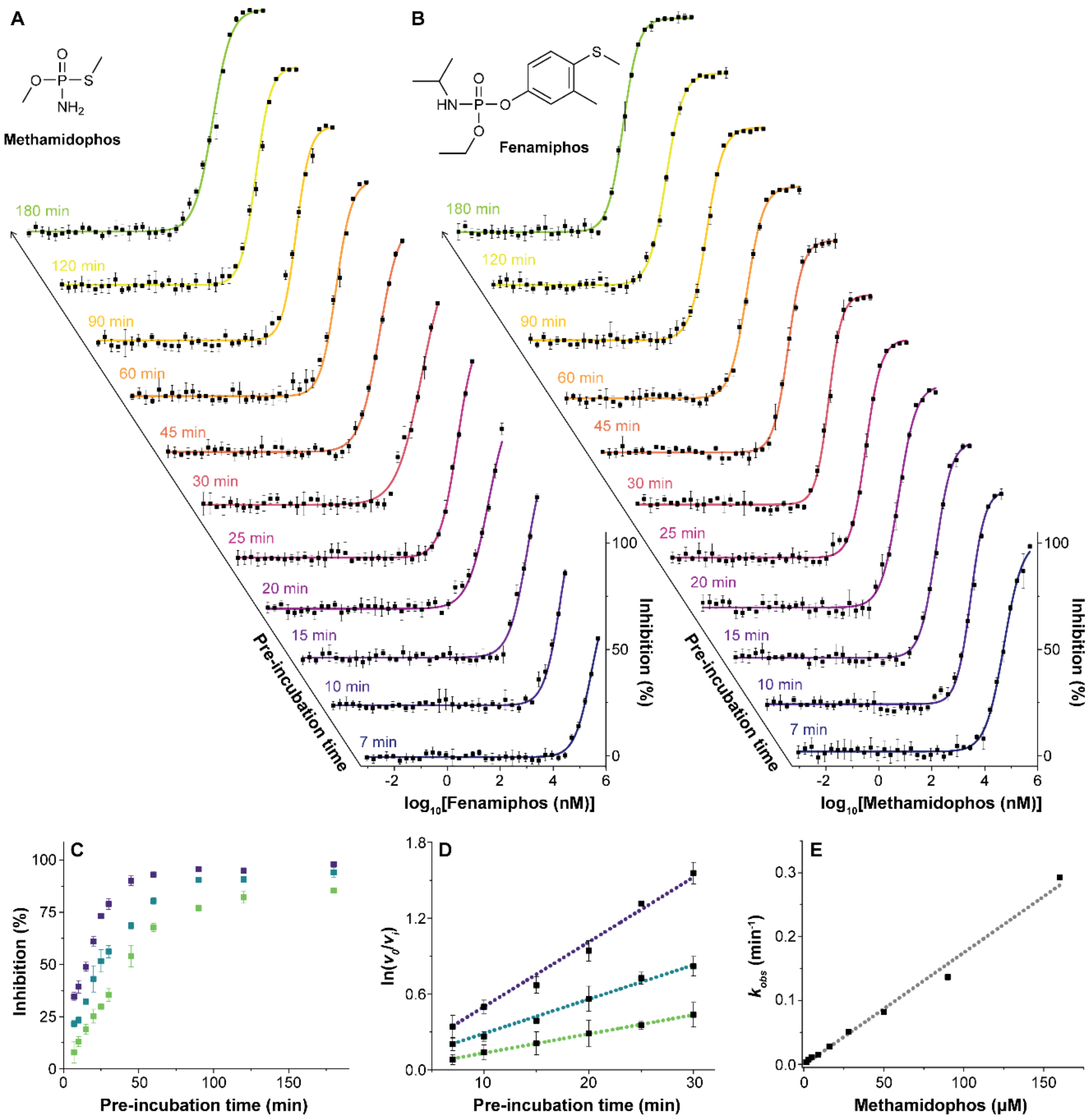

Fig. S5. Covalent inhibition of *h*AChE by fenamiphos and methamidophos monitored over a wide range of pre-incubation times and pesticide concentrations (**A** and **B**). Representative selection of inhibition across three methamidophos concentrations (top to bottom: 28, 16 and 9 uM) over the complete time course (**C**) as well as the corresponding normalized initial reaction rates (**D**) are also included. Calculated k_{obs} values (slopes of lines in **D**) were used for estimation of inhibition rate constants (k_i, slope of **E**). Identical analysis was done for both pesticides and AChEs. Error bars represent standard deviations (**A**-**D**) or standard errors from minimum least square estimations (**E**). The structures of both organophosphorus pesticides are also included.

High-throughput electrospray ionization mass spectrometry (HT-ESI-MS)

Experimental conditions. The injection volume for all samples was 1 µL. MS acquisition was performed using heated electrospray ionization (ESI) in the positive ion mode. Full scan mass analysis was carried out from *m/z* 100-150. The exact masses of acetylcholine (*m/z* 146.1176) and choline (*m/z* 104.1070) were monitored in all cases. Analysis was carried out automatically from up to four quenched and diluted 96-well plates sealed with silicon mats (Thermo Fisher Scientific, Waltham, MA) and located in the temperature-regulated (4 °C) autosampler compartment of the LC system. An Xcalibur sequence was created for the automated analysis, with independent RAW files being generated for each sample.

Data processing. All the RAW files were converted to mzXML using MSConvert from ProteoWizard (30), and then automatically processed using a custom MATLAB app. From all files, conversion ratios (*see* **Equation S1**) across each scan were calculated and averaged across a 2.5-second window (elution time: 1- 3.5 s) where the analyte elution was consistently observed (**Fig. S6A**).

Calibration. Calibration of the measured conversion ratios to obtain the absolute concentrations of choline produced by the reactions proceeded in a similar manner as that described for the UHT-DESI-MS assays. A 96-well plate was prepared using various proportions of 1 mM acetylcholine (Sigma Aldrich, St. Louis, MO) and 1 mM choline (Sigma Aldrich, St. Louis, MO) in the bioassay matrix (phosphate buffer 0.1 M pH 8 with 0.1% BSA) to obtain calibration standards (with a total analyte concentration of 1 mM) in triplicate. Only concentration ratios up to 30% were utilized as only initial velocities (i.e., low amounts of product generated) were of interest in the experiments. Note in this case a single overall concentration is used as the concentration of acetylcholine used as substrate in all experiments was fixed at 1 mM. The calibration curve is shown in **Fig. S6B**.

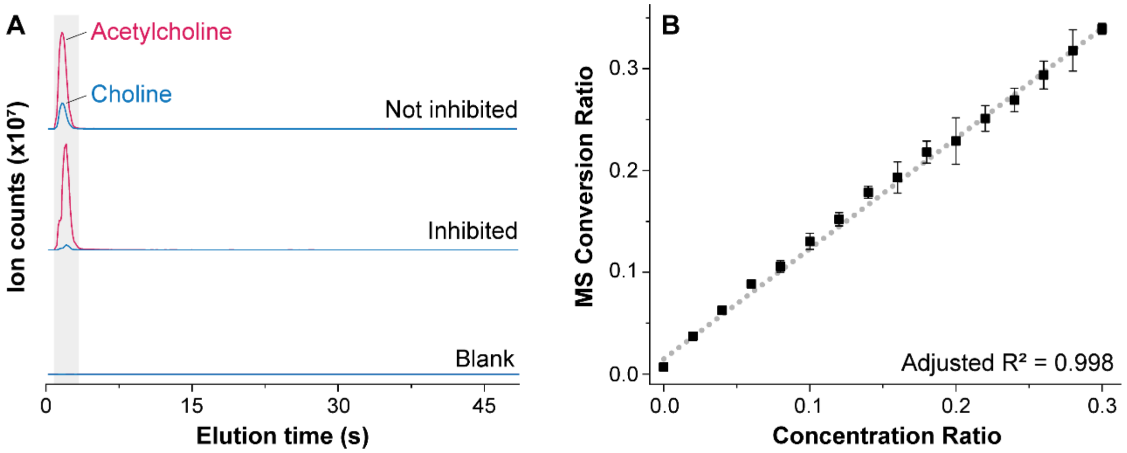

Fig. S6. Calibration and representative raw data from the HT-ESI-MS methodology. Calculated MS conversion ratios were converted to absolute concentration ratios using the calibration curve shown in **B**. Each data point represents the average of three independent replicates and error bars represent standard deviations. Conversion ratios were calculated by averaging the ion ratios calculated in scans between 1 and 3.5 s elution time. This window is shown in **A** as a shaded region over representative extracted ion currents for acetylcholine and choline from reactions in the absence (*top*) and presence (*middle*) of CWA. Blanks (*bottom*) were run throughout the plate sequences to verify the absence of carryover achieved with the method conditions.

Characterization of A-series inhibition of human acetylcholinesterase

A-series CWA synthesis. Synthesis, purification, and final analysis of A-230 [*N*-(1-(diethylamino)ethylidene)-*P*-methylphosphonamidic fluoride], A-232 [methyl (1-(diethylamino)ethylidene)-phosphoramidofluoridate], and A-234 [ethyl (1-(diethylamino)ethylidene)-phosphoramidofluoridate] were performed at LLNL, using in-house methods. HRMS (ESI/Q-TOF) analysis along with multi-nuclear (^{1}H, ^{13}C, ^{31}P, and ^{19}F) NMR experiments, however, demonstrated successful synthesis and purification of the CWA material with purities for all three compounds in excess of 97%.

Experimental details. Two series of 96-well plates were prepared using two different dilution series of A-230, A-232, or A-234 (all originating from 100 µg/mL stocks in methanol) prepared manually in phosphate buffer (100 mM pH 8.0). A low-concentration dilution series (0.3, 0.4, 0.5, 0.6, 0.9, 1.1, 1.4, 1.8, 2.4, and 3 ng/mL) was used for pre-incubation inhibition assays, whereas a high-concentration series (1, 2, 10, 15, 20, 30, 40, 100, 150, and 200 ng/mL) was used for competitive inhibition assays. Separate plates were prepared for each CWA and assay combination, with all dilution series including two agent-free controls. The final concentration of methanol in the assay mixtures was kept at 2%. All A-series inhibition assays used *h*AChE (Sigma Aldrich, St. Louis, MO). The enzyme was suspended in phosphate buffer (100 mM pH 8) with 0.1% BSA (Sigma Aldrich, St. Louis, MO) to a stock concentration of 1 µg/mL.

Pre-incubation assay. Following a similar methodology to that described for the UHT-DESI-MS-based characterization of pesticides inhibition, we attempted the characterization of the A-series agents following a pre-incubation methodology. Agent solutions were automatically mixed 1:1 with enzyme (final concentration: 250 ng/mL) to start the pre-incubation and aliquoted. To each aliquot, acetylcholine was added at different times (170-s intervals up to ca. 21 min) to a final concentration of 1 mM to start the reaction. After 2 minutes an aliquot of each reaction was quenched in acetonitrile (1:1000 dilution). Initial experiments (0.5-100 ppb CWA) showed too high potency from the agents, thus a lower concentration range of 0.15-1.5 ppb was utilized. All agent curves (i.e., 10 concentrations, 8 time points) were prepared in triplicate and analyzed via HT-ESI-MS. The results obtained for the k_i estimation (*see* **Equations S5** and **S6**) for each A-series agent are shown in **Fig. S7**. The obtained results are summarized in **Table 1** within the manuscript. Linear relationships were observed between the calculated k_{obs} and agent concentration, however, under the experimental conditions described ,the assumption of first-order kinetics might be compromised due to the low concentration of agents were required (despite previous literature (27) supporting the validity of the method after diluting high-potency agents). For this reason, we performed an independent set of experiments with a competition (i.e., substrate co-incubation) described below.

Co-incubation assay. This method allows for larger larger (~100X) agent concentrations to be used. In this case, acetylcholine and the A-series agents were initially mixed before the addition of the enzyme. All the additions made were of equal volumes, thus stock solution concentrations were three-fold higher compared to the final assay (1 mM and 125 ng/mL for acetylcholine and *h*AChE, respectively). After mixing, aliquots of each reaction mixture were automatically quenched in acetonitrile (1:1000 dilution) every 30 seconds starting 40 seconds after the enzyme addition for a total of ca. 4 min of reaction time. Note accurate, reproducible fast quenching across experiments is possible due to the use of an automated fluid handling system. Four independent replicates of each agent curve (i.e., 10 concentrations, 8 time points) were prepared and analyzed via HT-ESI-MS. The observed rate constant (k_{obs}) at each inhibitor concentration was estimated using **Equation S7** which correlates the assay response (R = choline produced through the reaction) with k_{obs} and the reaction time (t) using the initial rate of the uninhibited enzyme (v_i) as fixed parameter (25). Note the simplification due to the null reaction rate for the fully inhibited enzyme (v_s) and background (R_0, which was minimal and subtracted from the response before fitting). The agent-free controls were used for the calculation of v_i and the experimental conditions selected guaranteed linearity of this reaction during the time course monitored. The data obtained for A-230, A-232, and A-234 is shown in **Fig. S8A**, **S8C**, and **S8E**, respectively. Non-linear curve fitting was carried out using OriginPro 2023b. The calculated k_{obs} values were then utilized to estimate the

bimolecular inhibition rate constant, k_i, via linear regression using **Equations S8**, **S9**, and **S10**, as previously reported (18, 31–33). The estimated K_M for *h*AChE obtained in the UHT-DESI-MS characterization and the substrate concentration used in the competitive assay (*[S]*, 1 mM) were used as input where needed. Six concentrations (2.5 – 20 ppb) for each agent were used for k_i estimation, as lower concentrations showed no significant inhibition (compared to the unexposed control), and larger concentrations showed full inhibition of the enzyme through most of the time course studied, hindering the adequate estimation of k_{obs}. The linear plots following **Equation S8** are shown in **Fig. S8B**, **S8D**, and **S8F** for A-230, A-232, and A-234, respectively. The k_i results obtained are presented in **Table 1** within the manuscript. Additionally, it is worth noting using the model fit to the HT-ESI-MS data provides estimates of the dissociation constant (K_D) and the unimolecular bonding rate constant (k_2) of the inhibition process, as it considers it to proceed in two steps (i.e., reversible complex formation followed by irreversible adduction) (18, 31). As shown in **Table S3**, these values indicate the differences in inhibitory potency across toxic organophosphates roughly follow differences in affinity (i.e., K_D) rather than in reaction rate (k_2), something previously suggested for traditional agents (31). Note, as discussed in the manuscript for the k_i values, the estimated K_D and k_2 values are comparable to those of traditional CWAs such as VX and VR (18).

$$R = v_s t + \frac{v_i - v_s}{k_{obs}}[1 - e^{-k_{obs}t}] + R_0 = \frac{v_i}{k_{obs}}[1 - e^{-k_{obs}t}] \qquad \textbf{(Equation S7)}$$

$$\frac{1}{k_{obs}} = \frac{K_d}{k_2}\left(\frac{1}{[OP](1-\alpha)}\right) + \frac{1}{k_2} \qquad \textbf{(Equation S8)}$$

$$k_i = \frac{k_2}{K_d} \qquad \textbf{(Equation S9)}$$

$$\alpha = \frac{[S]}{K_M + [S]} \qquad \textbf{(Equation S10)}$$

Table S3. Estimated constants for the inhibition reaction of *h*AChE by A-series CWA using the competition method (i.e., substrate co-incubation). In all cases the standard errors from minimum least square estimation of the parameters (or via appropriate error propagation in the case of K_D, which is calculated using Equation S9) are shown.

**A-series agent**	**K_D (M)**	**k_2 (min^{-1})**	**k_i (M^{-1} min^{-1})**
A-230	(5.0 ± 0.7) x 10^{-8}	4.2 ± 0.6	(8.3 ± 0.2) x 10^{7}
A-232	(9 ± 2) x 10^{-8}	4 ± 1	(4.5 ± 0.1) x 10^{7}
A-234	(8 ± 2) x 10^{-8}	4 ± 1	(4.8 ± 0.1) x 10^{7}

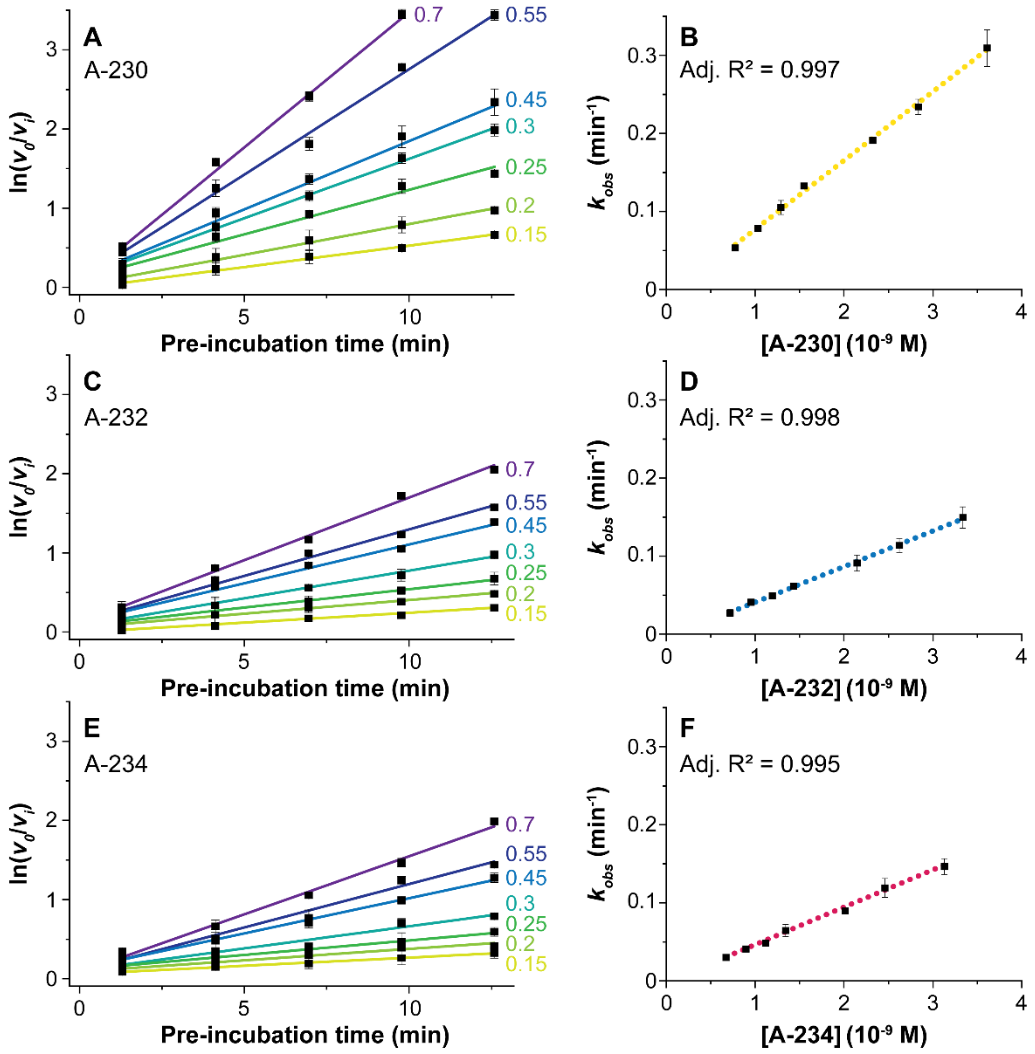

Fig. S7. Inhibition of *h*AChE by A-series CWA studied using a pre-incubation methodology. The linear relationships between the normalized initial reaction rates and the pre-incubation time for different CWA concentrations (**A**, **C**, **E**; denoted concentrations are in ppb) are included, as well as those between the observed rate constants and the agent concentration (**B**, **D**, **F**). These latter are utilized for estimation of inhibition constants (from the estimated slopes). Error bars indicate standard deviations (**A**, **C**, **E**; n = 3) or estimated standard errors from errors from minimum least square estimations (**B**, **D**, **F**).

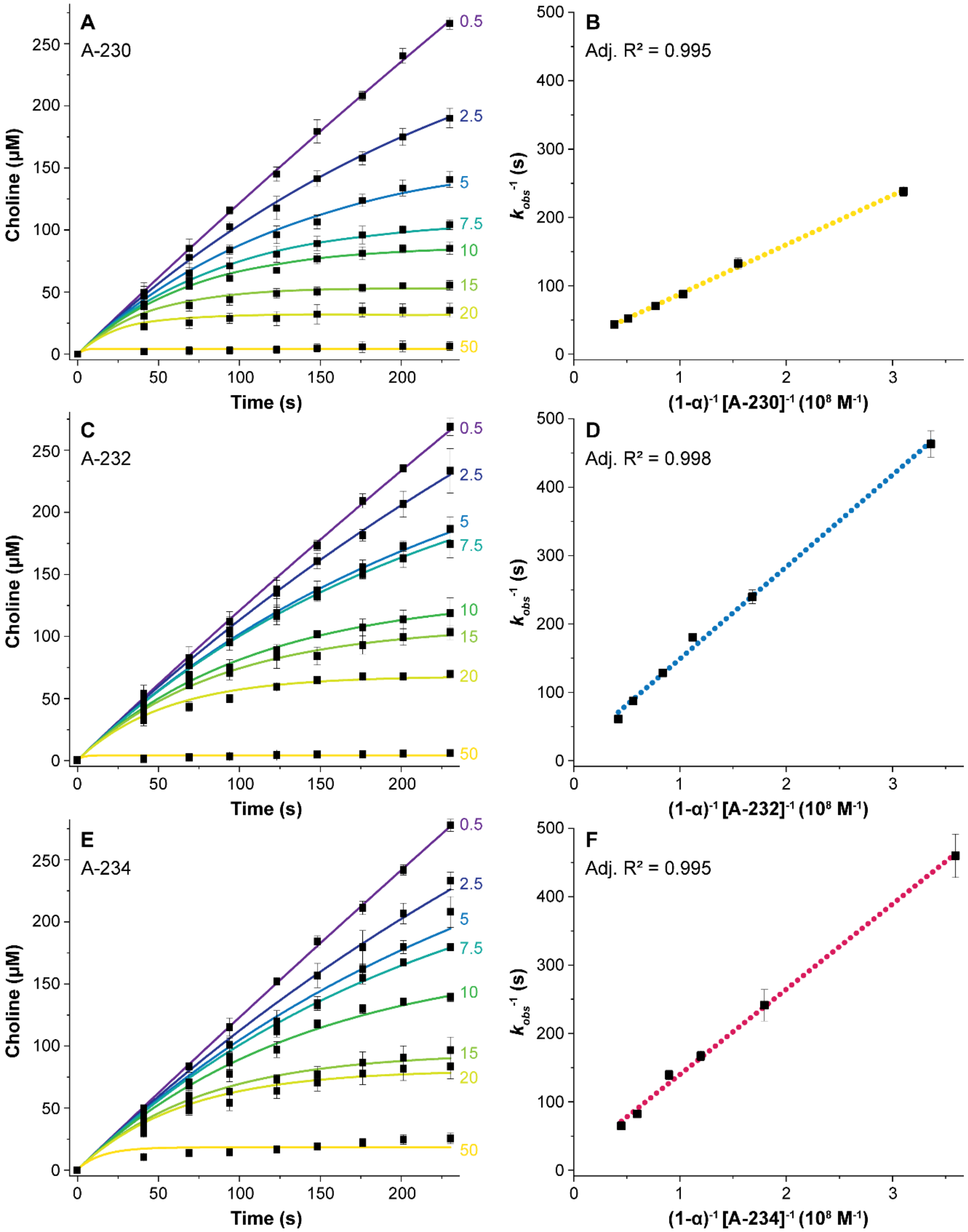

Fig. S8. Inhibition of *h*AChE by A-series CWA studied using a competition methodology (i.e., substrate co-incubation). The progress curves obtained for all agents (**A**, **C**, **E**; denoted concentrations are in ppb) are included, as well as the plots showing the linear relationship between the observed rate constants and the agent concentration, which are utilized for estimation of inhibition constants (**B**, **D**, **F**). Error bars indicate standard deviations (**A**, **C**, **E**; n = 4) or estimated standard errors from non-linear curve fitting (**B**, **D**, **F**).

Generation, purification and evaluation of adducted human cholinesterase for multi-facility studies

Synthesis of NEMP. NEMP (4-nitrophenyl ethyl methylphosphonate) was synthesized, purified and characterized using methodologies previously reported (34).

Control experiments. Adduction, purification, and transportation processes were carried out for A-series adducted AChE, as well as for unadducted and VX/NEMP-adducted enzyme. Potential functional alterations due to the enzyme processing were evaluated by comparing the kinetic behavior of the shipped unadducted enzyme and the equivalent solution prepared and characterized in advance (*see* section on *Label-free characterization of cholinesterases*, pages 5-8). Enzyme activity was found to be identical in both cases, indicating that the process does not significantly affect enzyme function. Similarly, the VX and NEMP reactivation experiments showed the expected behavior for previously characterized oximes, indicating no effect of the purification process in the adduct reactivation.

LC-MS/MS quantitation of free agents. A Thermo Scientific Vanquish Flex HPLC with a Thermo Scientific Q Exactive™ HF-X Orbitrap™ MS was used to monitor A-series CWA concentration post-ChE exposure. The LC column used was a Waters ACQUITY HSS T3 (2.1 x 150 mm, 1.8 µm). The mobile phase consisted of a gradient elution, where solvent A = Milli-Q H_2O with 0.1% formic acid and solvent B = acetonitrile with 0.1% formic acid. The solvent gradient was 15 min in length: initial 1% B for 2 min, ramp to 95% B over 7 min, hold at 95% B for 1 min, and re-equilibrate at 1% B for 5 min. The flow rate was 0.25 mL/min. The injection volume for all samples was 2 µL, and the column oven temperature was maintained at 40 °C. MS acquisition was performed using heated ESI in positive mode. This MS experiment was composed of a full scan (*m/z* 75-750) analysis combined with targeted PRM analysis. For A-230, A-232, and A-234 the monitored parent $[M+H]^+$ ions were *m/z* 195.1057, 211.1006, and 225.1163, respectively, using a 1.0 *m/z* isolation window and a collision energy (CE) of 18 for all three small molecules. Ultra-high purity nitrogen served as the collision gas. Using this methodology, we validated that the level of free agent in any of the samples shipped for UHT reactivation screening studies were safe for handling in a standard laboratory facility. In all cases, the concentration of agent achieved was sub ng/mL, significantly below reported median lethal dose (LD_{50}) values for CWAs.

Evaluation of aging of A-series adducted cholinesterases

LC-MS/MS analysis of *eq*BChE digestion. A Thermo Scientific Vanquish Flex HPLC with a Thermo Scientific Q Exactive™ HF-X Orbitrap™ MS was used for the peptide analysis. The LC column used for the analysis was an Agilent AdvanceBio Pepide Mapping (2.1 x 150 mm, 2.7 µm) equipped with an AdvanceBio Peptide Mapping (2.1 x 5 mm, 2.7 µm) guard column. The mobile phase consisted of a gradient elution, where solvent A = Milli-Q H_2O with 0.1% formic acid and solvent B = acetonitrile with 0.1% formic acid. The solvent gradient was 20 min in length: initial 5% B for 0.5 min, ramp to 35% B over 9.5 min, ramp to 95% B over 2 minutes, hold at 95% B for 3 minutes, and re-equilibrate at 5% B for 5 min. The flow rate was 0.3 mL/min. The injection volume for all samples was 10 µL, and the column oven temperature was maintained at 40 °C. MS acquisition was performed using heated electrospray ionization (ESI) in the positive ion mode. Ultra-high purity nitrogen served as the collision gas. This LC-MS/MS experiment was composed of a full scan (*m/z* 100-1500) analysis combined with targeted parallel reaction monitoring (PRM) analysis. **Table S4** below summarizes the adducted and unadducted peptides monitored for the aging study. Specific peptide retention time (RT) standards (Pierce™ Peptide Retention Time Calibration Mixture, Thermo Fisher Scientific, 88320) were chosen to roughly span the expected RT range for the targeted nonapeptides.

Aging results. The results of the aging experiments carried out are summarized in **Fig. S9**. As shown, good stability (i.e. no significant aging) is observed on the adducted nonapeptide over a

one-month period. These data were used to ensure that the A-series exposed ChE material would not age significantly during transit, as aged adducts are well known to not reactivate. Nevertheless, material transfer was coordinated to minimize time between sample preparation (LLNL) and reactivation screening (Purdue).

Table S4. Nonapeptide species of interest targeted in the parallel reaction monitoring (PRM) LC-MS/MS experiments. **Adducted nonapeptide = nominal nonapeptide m/z + 174.0922, 190.0871, or 204.1027 for A-230, A-232, or A-234, respectively. **Aged nonapeptide = nominal nonapeptide m/z + 77.9870, 93.9820, or 107.9976 for A-230, A-232, or A-234, respectively.*

**Peptide Identity**	**Sequence**	**PRM *m/z***	**RT window (min)**
*h*BChE native nonapeptide	FGESAGAAS	796.3472	3.5 – 4.5
A-230-*h*BChE nonapeptide	FGES*AGAAS	970.4394	6.1 – 7.1
A-232-*h*BChE nonapeptide	FGES*AGAAS	986.4343	6.5 – 7.5
A-234-*h*BChE nonapeptide	FGES*AGAAS	1000.4499	7.1 – 8.1
A-230-*h*BChE nona. w/ amidine loss	FGES**AGAAS	874.3342	n.d.
A-232-*h*BChE nona. w/ amidine loss	FGES**AGAAS	890.3292	n.d.
A-234-*h*BChE nona. w/ amidine loss	FGES**AGAAS	904.3448	n.d.
RT Peptide #3 $[M + 2H]^{2+}$	HVLTSIGEK(heavy)	496.2867	4.0 – 5.0
RT Peptide #12 $[M + 2H]^{2+}$	LTILEELR(heavy)	498.8018	8.3 – 9.3

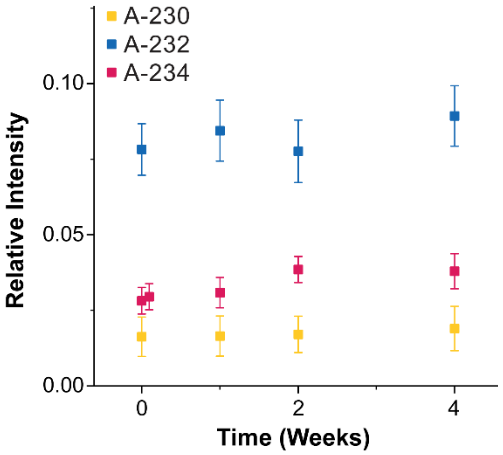

Fig. S9. Aging results for adducted *eq*BChE over a one-month period. The results represent the ratio between the adducted, unaged *eq*BChE nonapeptide response relative to that of the negative control (i.e., unexposed *eq*BChE nonapeptide sample) over time. Relative intensities are plotted to account for inter-day analysis variability. Error bars represent estimated measurement uncertainty using technical replicates.

Synthesis and characterization of oxime candidates

Chemicals, reagents, and supplies. All chemicals were purchased from commercial suppliers and used as received. 1-(Hydroxyimino)propan-2-one, ethylenediamine, 1,3-diaminopropane, and 1-Boc-ethylenediamine were purchased from Combiblocks (San Diego, CA.). Anhydrous ethanol, dichloromethane, 4-methoxybenzylamine, 4-chlorobenzylamine, benzylamine, aniline, 1,4-bis(aminomethyl)-benzene along with the pyridinium oximes: MMB-4 (***32***, bromide salt), 2-PAM (***29***, chloride salt), HI-6 (***30***, chloride salt) and Obidoxime (***31***, chloride salt) were purchased from Sigma-Aldrich (St. Louis, MO.). 1-(2-Aminoethyl)piperidine and *N*-(2-aminoethyl)morpholine were purchased from TCI America (Portland, OR.). Sodium bicarbonate, anhydrous sodium sulfate, and sodium chloride were purchased from Acros Organics (Westchester, PA.). Deuterated chloroform ($CDCl_3$) and DMSO-d_6 were purchased from Cambridge Isotope Laboratories (Tewksbury, MA). Solvents used during the syntheses were removed by using a Büchi rotary evaporator R-200 equipped with a Büchi heating bath B-490 and coupled to a KNF Laboport Neuberger UN820 vacuum pump. Autosampler vials and glass inserts used for GC-MS and LC-MS analyses were purchased from Agilent Technologies (Santa Clara, CA.). Wheaton scintillation vials (20 mL capacity) were purchased from VWR (Radnor, PA).

Analytical methods. ^{1}H NMR (600 MHz) and ^{13}C NMR (151 MHz) were recorded in either $CDCl_3$ or DMSO-d_6. Spectra were obtained using a Bruker Avance III 600 MHz instrument equipped with a Bruker TCI 5 mm cryoprobe (Bruker Biospin, Billerica, MA) at 30.0 ± 0.1 ˚C. ^{1}H NMR chemical shifts are calibrated with respect to residual chloroform in $CDCl_3$ centered at 7.26 ppm or DMSO-d_6 centered at 2.54 ppm, whereas for ^{13}C NMR, the center peak for $CDCl_3$, centered at 77.0 ppm or DMSO-d_6 centered at 40.5 ppm, were used for the calibration. In some ^{13}C spectra the specific assignment of a given carbon (e.g., C=O) to a signal was done with the aid of ^{13}C DEPT-135. Hybrid direct infusion of oxime reference standards was performed by injecting 5 µL of a 1000 ppm reference into an Agilent 6530 Accurate Mass QTOF coupled to an Agilent 1290 Infinity UHPLC flowing 5 mM ammonium acetate in 50% aqueous acetonitrile at a flow rate of 0.25 mL/min. High-resolution protonated precursor ions with a mass error of less than 10 ppm for the compounds of interest were identified in each of the individual reference standards.

Synthesis of N-substituted 2-hydroxyiminoacetamidine oximes (oximes ***1*** through ***14***). These oximes were prepared and purified as previously described by the Sharpless and Taylor groups (35, 36) as well as our group (37, 38).

Synthesis and characterization of MINA-based oximes (oximes ***15*** through ***24***). 1-(Hydroxyimino)propan-2-one (monoisonitrosoacetone, MINA; 100 mg, 1.15 mmol) was dissolved in ethanol (EtOH, 5 mL) in a 20 mL scintillation vial equipped with a stir bar. To the light brown solution, the corresponding amine (1.15 mmol, 1.0 equiv. to MINA or 0.58 mmol for diamine species) was added in a single portion, and the resulting solution was stirred at ambient temperature overnight. In all cases, a precipitate formed overnight, which was isolated via filtration and washed with cold EtOH (3 x 3 mL). The solid was placed under vacuum to remove any residual EtOH.

Oxime ***15***: Collected as a light beige solid (158 mg, 67%). ^{1}H NMR (DMSO-d_6, 600 MHz) □ 11.63 (s, 1H), 7.57 (s, 1H), 7.23 (d, J = 8.7 Hz, 2H), 6.87 (d, J = 8.7 Hz, 2H), 4.51 (s, 2H), 3.71 (s, 3H), 2.07 (s, 3H); ^{13}C NMR (DMSO-d_6, 150 MHz) δ 163.9, 158.4, 152.3, 132.6, 129.4, 114.1, 55.5, 54.9, 14.0. HRMS (ESI/Q-TOF) *m/z*: $[M+H]^+$ calculated for $C_{11}H_{15}N_2O_2$ 207.1128; found 207.1134.

Oxime ***16***: Collected as an off-white solid (145 mg, 72%). ^{1}H NMR (DMSO-d_6, 600 MHz) □ 11.66 (s, 1H), 7.59 (s, 1H), 7.32 (m, 4H), 7.22 (m, 1H), 4.59 (s, 2H), 2.08 (s, 3H); ^{13}C NMR (DMSO-d_6, 150 MHz) δ 163.9, 151.8, 140.2, 128.3, 127.8, 126.5, 55.0, 13.6. HRMS (ESI/Q-TOF) *m/z*: $[M+H]^+$ calculated for $C_{10}H_{13}N_2O$ 177.1022; found 177.1016.

Table S5. Oxime panel screened for reactivation activity towards CWA-adducted *h*AChE. The majority of this panel was developed as potential blood brain barrier-penetrating oximes, with lead candidate ***12*** (LLNL-02) being previously developed and evaluated by members of this group **(37)**. Four established oximes were included in the panel: ***29*** = 2-PAM (pralidoxime), ***30*** = HI-6 (asoxime), ***31*** = obidoxime, ***32*** = MMB-4. To our knowledge this report is the first time imineoxime candidates ***15*** through **24** have been evaluated as reactivators for CWA exposure.

Oxime code	Structure	Oxime code	Structure	Oxime code	Structure
1	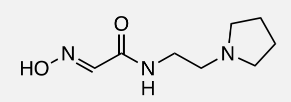	12	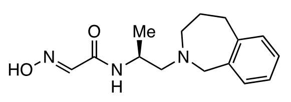	23	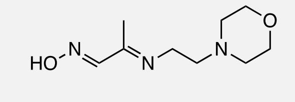
2	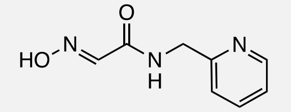	13	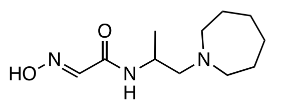	24	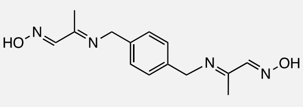
3	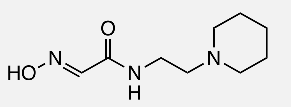	14	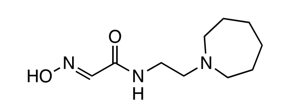	25	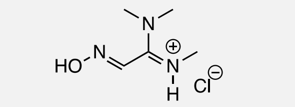
4	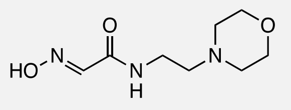	15	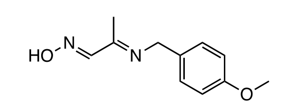	26	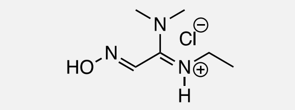
5	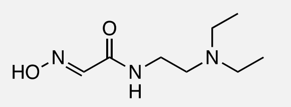	16	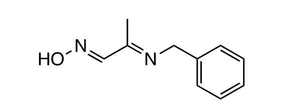	27	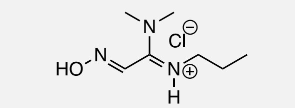
6	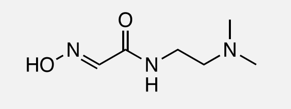	17	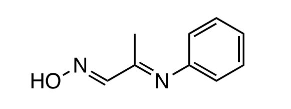	28	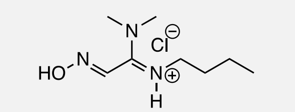
7	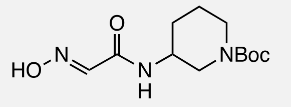	18	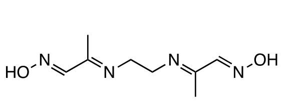	29	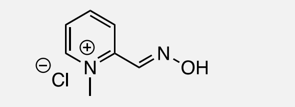
8	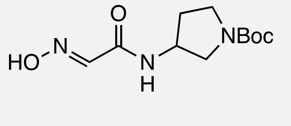	19	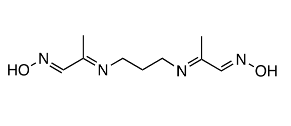	30	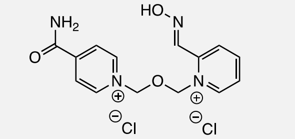
9	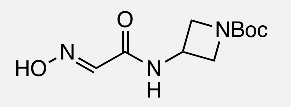	20	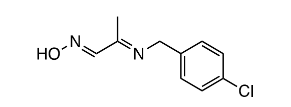	31	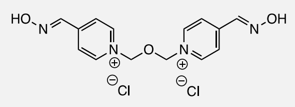
10	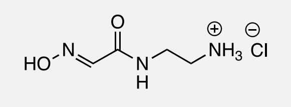	21	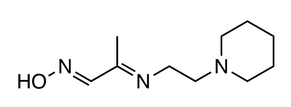	32	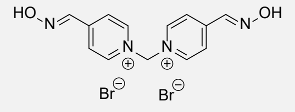
11	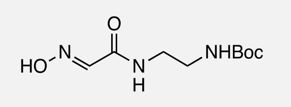	22	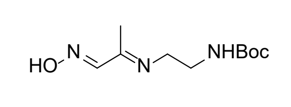		

Oxime ***17***: Collected as a light brown solid (71 mg, 38%). Dominant diastereomer: ^{1}H NMR ($CDCl_3$, 600 MHz) □ 11.98 (s, 1H), 7.74 (s, 1H), 7.33 (m, 2H), 7.08 (m, 1H), 6.75 (m, 2H), 1.92 (s, 3H); ^{13}C NMR (DMSO-d_6, 150 MHz) δ 163.8, 151.2, 150.0, 129.0, 123.8, 119.2, 15.3. Lesser diastereomer: ^{1}H NMR ($CDCl_3$, 600 MHz) □ 12.1 (s, 1H), 7.40 (s, 1H), 7.31 (m, 2H), 7.08 (m, 1H), 6.72 (m, 2H), 2.26 (s, 3H); ^{13}C NMR (DMSO-d_6, 150 MHz) δ 163.9, 150.0, 142.5, 129.1, 123.8, 119.5, 22.4. Diastereomer ratio 5.3:1 (measured by ^{1}H NMR). HRMS (ESI/Q-TOF) *m/z*: $[M+H]^+$ (single peak) calculated for $C_9H_{11}N_2O$ 163.0866; found 163.0872.

Oxime ***18***: Collected as a white solid (48 mg, 42%). ^{1}H NMR (DMSO-d_6, 600 MHz) □ 11.59 (s, 2H), 7.50 (s, 2H), 3.65 (s, 4H), 1.98 (s, 6H); ^{13}C NMR (DMSO-d_6, 150 MHz) δ 163.8, 151.7, 52.3, 13.4. HRMS (ESI/Q-TOF) m/z: $[M+H]^+$ calculated for $C_8H_{15}N_4O_2$ 199.1190; found 199.1197.

Oxime ***19***: Collected as a white solid (66 mg, 54%). ^{1}H NMR (DMSO-d_6, 600 MHz) □ 11.56 (s, 2H), 7.52 (s, 2H), 3.41 (t, *J* = 6.9 Hz, 4H), 1.95 (s, 3H), 1.91 (m, J = 6.9 Hz, 2H); ^{13}C NMR (DMSO-d_6, 150 MHz) δ 163.6, 152.2, 49.5, 31.6, 13.6. HRMS (ESI/Q-TOF) *m/z*: $[M+H]^+$ calculated for $C_9H_{17}N_4O_2$ 213.1346; found 213.1333.

Oxime ***20***: Collected as a light beige solid (133 mg, 55%). ^{1}H NMR (DMSO-d_6, 600 MHz) □ 11.67 (s, 1H), 7.59 (s, 1H), 7.37 (br, 4H), 4.57 (s, 2H), 2.08 (s, 3H); ^{13}C NMR (DMSO-d_6, 150 MHz) δ 164.7, 152.1, 139.7,131.4, 130.0, 128.6, 54.5, 14.1. HRMS (ESI/Q-TOF) *m/z*: $[M+H]^+$ calculated for $C_{10}H_{12}ClN_2O$ 211.0633; found 211.0623.

Oxime ***21***: Collected as an off-white solid (143 mg, 63%). ^{1}H NMR (DMSO-d_6, 600 MHz) □ 11.57 (s, 1H), 7.50 (s, 1H), 3.46 (t, *J* = 7.4 Hz, 2H), 2.50 (t, *J* = 7.4 Hz, 2H), 2.35 (br, 4H), 1.96 (s, 3H), 1.45 (m, 4H), 1.35 (m, 2H); ^{13}C NMR (DMSO-d_6, 150 MHz) δ 163.8, 152.2, 59.6, 54.8, 49.9, 26.0, 24.5, 13.6. HRMS (ESI/Q-TOF) *m/z*: $[M+H]^+$ calculated for $C_{10}H_{20}N_3O$ 198.1601; found 198.1601.

Oxime ***22***: Collected as an off-white solid (134 mg, 51%). ^{1}H NMR (DMSO-d_6, 600 MHz) □ 11.60 (s, 1H), 7.51 (s, 1H), 3.39 (br m, 2H), 3.16 (br m, 2H), 1.94 (s, 3H) 1.35 (s, 9H); ^{13}C NMR (DMSO-d_6, 150 MHz) δ 164.4, 156.0, 152.1, 78.0, 51.5, 41.2, 28.7, 13.7. HRMS (ESI/Q-TOF) *m/z*: $[M+H]^+$ calculated for $C_{10}H_{20}N_3O_3$ 230.1499; found 230.1510.

Oxime ***23***: Collected as a light beige solid (137 mg, 60%). ^{1}H NMR (DMSO-d_6, 600 MHz) □ 11.56 (s, 1H), 7.52 (s, 1H), 3.56 (t, J = 4.7 Hz, 4H), 3.50 (t, J = 7.1 Hz, 2H), 2.57 (t, J = 7.1 Hz, 2H), 2.42 (b, 4H), 1.98 (s, 3H); ^{13}C NMR (DMSO-d_6, 150 MHz) δ 163.4, 151.7, 66.2, 58.8, 53.6, 49.2, 13.2. HRMS (ESI/Q-TOF) *m/z*: $[M+H]^+$ calculated for $C_9H_{18}N_3O_2$ 200.1394; found 200.1398.

Oxime ***24***: Collected as a beige solid (65 mg, 41%). ^{1}H NMR (DMSO-d_6, 600 MHz) □ 11.65 (s, 2H), 7.58 (s, 2H), 7.28 (s, 4H), 4.57 (s, 4H), 2.08 (s, 6H); ^{13}C NMR (DMSO-d_6, 150 MHz) δ 163.8, 151.8, 138.4, 127.8, 54.8, 13.6. HRMS (ESI/Q-TOF) m/z: $[M+H]^+$ calculated for $C_{14}H_{19}N_4O_2$ 275.1503; found 275.1514.

Synthesis of amidine oximes (oximes ***25*** through ***28***). These oximes were prepared as previously described by the Cashman group (39).

Ultrahigh-throughput screening of oxime candidates as reactivators

Experimental details for panel screening. Stock oxime solutions were prepared in DMSO at 50 mM concentrations guaranteeing less than 0.2% DMSO in the final assay solutions. Enzyme and oxime solutions were diluted in phosphate buffer (0.1 M pH 8.0) with 0.1% BSA, equilibrated at 37 °C, and mixed in a 1:1 ratio to obtain 20 µL of 10 ng/mL *h*AChE and various oxime concentrations. Mixtures were aliquoted and acetylcholine (2 µL, 10 mM; final assay concentration of 1 mM) was added to start the AChE reaction after predefined reactivation time points before quenching with ice-cold acetonitrile after 5 minutes. Samples were immediately analyzed via UHT-DESI-MS. Panel screening experiments explored three reactivation time points (30 minutes, 1 hour, and 4 hours) at a fixed reactivator dose (100 µM) against VX and NEMP. Dose-response confirmation experiments for selected oximes were conducted with a 4-hour reactivation time. Sigmoidal fitting of the dose-response curves was carried out using OriginPro 2023b, and half-maximum effective concentrations (EC_{50}) were estimated (**Table S6**), when appropriate. In all experiments, non-adducted enzyme controls were run and used for normalization (**Equation S11**), thus accounting for the known competitive inhibition of AChE at high oxime concentrations (typically ≥100 µM). Throughout screening, the highest inhibition observed (at 100 µM) was ~30%, with the average value across the panel being 4% and high consistency observed across reactivation times (i.e., competitive inhibition is independent of the pre-incubation time between the enzyme and the oxime). Importantly, due to the use of the native AChE substrate, acetylcholine, no oximolysis interferences were observed over a wide range of reactivator concentrations even after long co-incubation (up to 4 hours) of the reagents as shown in **Fig. S10** for a selection of oximes.

Experimental details for A-series reactivation assays. Established oximes (***29***-***32***) were screened as reactivators under the same conditions used for the VX and NEMP cases (i.e., 100 µM final concentrations and three reactivation times: 30 min, 1h, and 4 h). Confirmatory dose-response experiments were then conducted with a fixed 4-hour reactivation time varying the oxime concentration (1 nM – 1 mM). Experimental results are shown in **Fig. 4A**, **4B,** and **S11**. Lengthier reactivation attempts were conducted with the most promising oxime, HI-6 (***30***), as well as the seemingly unactive, but well-established 2-PAM (***29***). For this, mixtures of enzyme and oximes were prepared (final concentrations 10 ng/mL and 100 µM, respectively) and incubated for up to 72 hours. At defined time intervals 20-µL aliquots were taken from the reactions, spiked with 2 µL of acetylcholine solution (final concentration: 1 mM) and quenched with ice-cold acetonitrile after 5 minutes. Enzyme reactivation was monitored up to 72 hours, and non-adducted *h*AChE samples were subjected to an identical process to serve as experimental controls. No significant decrease in activity was observed in the non-adducted *h*AChE controls over the reactivation times monitored, and no significant spontaneous reactivation was observed in adducted *h*AChE controls in absence of oxime reactivators. The inhibition results showed modest reactivation by 2-PAM (***29***) even at long timescales whereas higher potencies were observed for HI-6 (***30***). The A-series-adducted *h*AChE reactivation results are summarized in **Table S7**.

Table S6. Estimated EC_{50} (µM) values for a selection of oximes with diverse reactivation potencies against VX- and NEMP-adducted *h*AChE as observed in the primary panel screen. Estimated standard errors are indicated in all cases. O.R. denotes out-of-range estimation, meaning that ≥50% reactivation was not observed within the concentration range explored.

Oxime	EC_{50} (µM)	
	VX	NEMP
2	398 ± 18	284 ± 99
3	3.5 ± 0.3	3.4 ± 0.1
11	O.R.	O.R.
12	22 ± 1	36 ± 3
29	0.78 ± 0.07	0.76 ± 0.07
30	0.59 ± 0.04	0.71 ± 0.01

$$Inhibition_{AChEOP+[Ox]}\ (\%) = \left(1 - \frac{CR_{AChEOP+[Ox]} - CR_{AChEOP}}{CR_{AChE+[Ox]} - CR_{AChEOP}}\right) 100\% \qquad \textbf{(Equation S11)}$$

$CR = Conversion\ Ratio;\ AChEOP = Adducted\ enzyme;\ AChE = Nonadducted\ enzyme\ control;\ [Ox] = Oxime\ at\ a\ specific\ concentration$

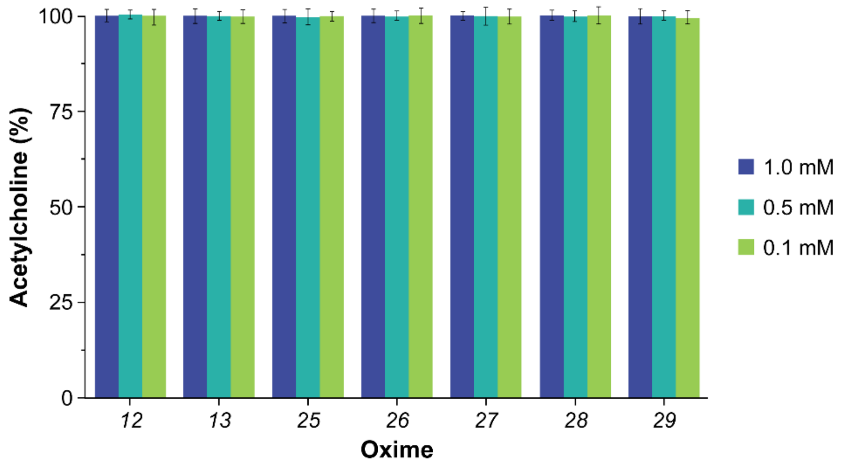

Fig. S10. Evaluation of oxime-induced hydrolysis of acetylcholine. Several oximes were studied at different concentrations as indicated in the color legend. As shown, no significant oxime-induced hydrolysis of acetylcholine (1 mM) occurs after incubation for 4 h at 37 °C. In all cases error bars represent standard deviations (n = 32).

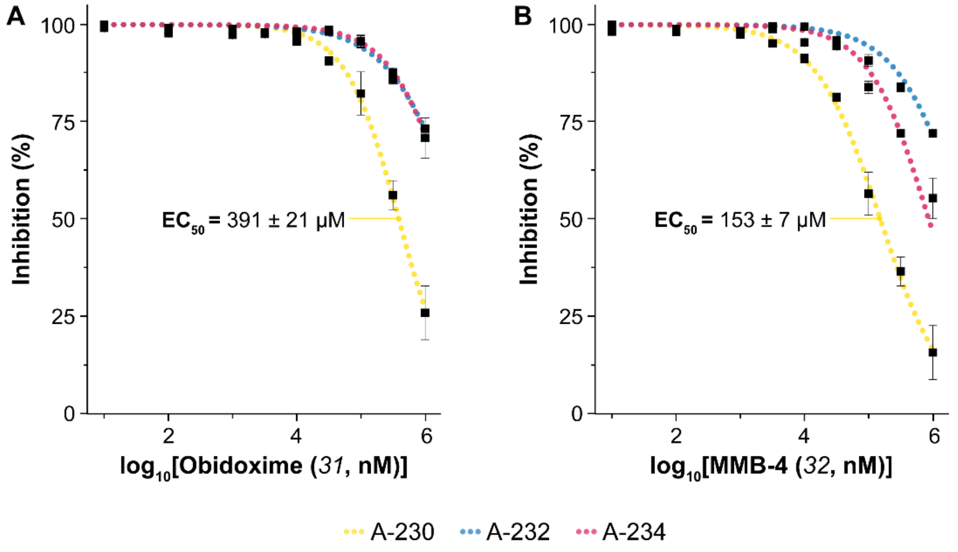

Fig. S11. Dose-response curves for the reactivation of A-series agents using established oximes ***31*** (**A**) and ***32*** (**B**). In this case the reactivation time was constant (4 h at 37 °C) while the oxime concentration varied. Inhibition values were normalized to positive controls using non-adducted *h*AChE in the presence of equivalent concentrations of oxime and negative controls in the absence of enzyme. For the most efficient reactivation cases, EC_{50} values with their associated standard errors were estimated through sigmoidal fitting and are indicated. In all cases error bars represent standard deviations (n = 16).

Table S7. Performance of established oxime reactivators against A-series-adducted *h*AChE. The table indicates the reactivation times necessary to reach ~50% normalized inhibition response using four established oxime reactivators (***29***-***32***; 100 µM). O.R. denotes out-of-range estimation, meaning that ≥50% reactivation was not observed within the time course explored as denoted in brackets. Note that long reactivation experiments (>4 h) were only conducted for oximes ***29*** and ***30*** (i.e. 2-PAM and HI-6). For the oximes showing significant reactivation of A-230-adducted *h*AChE after 4 h, estimated EC_{50} values are given with associated standard errors estimated through sigmoidal fitting. **Significant reactivation was observed after 4 h, but inhibition values below 50% were obtained using >100 µM concentrations as indicated by the estimated EC_{50} values.*

Oxime	A-230	A-230 4 h EC_{50} (µM)	A-232	A-234
29	O.R. (72 h)	N.A.	O.R. (72 h)	O.R. (72 h)
30	4 h	74 ± 2	72 h	36 h
31	4 h*	391 ± 21	O.R. (4 h)	O.R. (4 h)
32	4 h*	153 ± 7	O.R. (4 h)	O.R. (4 h)

SI References

1. N. M. Morato, M. T. Le, D. T. Holden, R. G. Cooks, Automated High-Throughput System Combining Small-Scale Synthesis with Bioassays and Reaction Screening. *SLAS Technol.* **26**, 555–571 (2021).
2. T. J. P. Sobreira, *et al.*, High-throughput screening of organic reactions in microdroplets using desorption electrospray ionization mass spectrometry (DESI-MS): hardware and software implementation. *Anal. Methods* **12**, 3654–3669 (2020).
3. N. M. Morato, D. T. Holden, R. G. Cooks, High-Throughput Label-Free Enzymatic Assays Using Desorption Electrospray-Ionization Mass Spectrometry. *Angew. Chem. Int. Ed.* **59**, 20459–20464 (2020).
4. G. L. Ellman, K. D. Courtney, V. Andres, R. M. Featherstone, A new and rapid colorimetric determination of acetylcholinesterase activity. *Biochem. Pharmacol.* **7**, 88–95 (1961).
5. J.-P. Colletier, *et al.*, Structural insights into substrate traffic and inhibition in acetylcholinesterase. *EMBO J.* **25**, 2746–2756 (2006).
6. E. Reiner, V. Simeon-Rudolf, Cholinesterase: substrate inhibition and substrate activation. *Pflüg. Arch. - Eur. J. Physiol.* **440**, R118–R120 (2000).
7. J. Shenouda, P. Green, L. Sultatos, An Evaluation of the Inhibition of Human Butyrylcholinesterase and Acetylcholinesterase by the Organophosphate Chlorpyrifos Oxon. *Toxicol. Appl. Pharmacol.* **241**, 135–142 (2009).
8. A. R. Mukhametgalieva, S. V. Lushchekina, A. R. Aglyamova, P. Masson, Steady-state kinetic analysis of human cholinesterases over wide concentration ranges of competing substrates. *Biochim. Biophys. Acta BBA - Proteins Proteomics* **1870**, 140733 (2022).
9. M. Naveh, Z. Bernstein, D. Segal, Y. Shalitin, New substrates of acetylcholinesterase. *FEBS Lett.* **134**, 53–56 (1981).
10. R. J. Kitz, Human tissue cholinesterases: Rates of recovery after inhibition by neostigmine; Michaelis-Menten constants. *Biochem. Pharmacol.* **13**, 1275–1282 (1964).
11. S. Cheng, Q. Wu, H. Xiao, H. Chen, Online Monitoring of Enzymatic Reactions Using Time-Resolved Desorption Electrospray Ionization Mass Spectrometry. *Anal. Chem.* **89**, 2338–2344 (2017).
12. C. V. Altamirano, C. F. Bartels, O. Lockridge, The Butyrylcholinesterase K-Variant Shows Similar Cellular Protein Turnover and Quaternary Interaction to the Wild-Type Enzyme. *J. Neurochem.* **74**, 869–877 (2000).
13. P. Masson, *et al.*, High activity of human butyrylcholinesterase at low pH in the presence of excess butyrylthiocholine. *Eur. J. Biochem.* **270**, 315–324 (2003).
14. A. Saxena, *et al.*, The pH Dependence of Dealkylation in Soman-Inhibited Cholinesterases and Their Mutants: Further Evidence for a Push−Pull Mechanism. *Biochemistry* **37**, 15086–15096 (1998).
15. J. C. Lee, J. A. Harpst, Purification and properties of butyrylcholinesterase from horse serum. *Arch. Biochem. Biophys.* **145**, 55–63 (1971).
16. D. Kaplan, *et al.*, Does "Butyrylization" of Acetylcholinesterase through Substitution of the Six Divergent Aromatic Amino Acids in the Active Center Gorge Generate an Enzyme Mimic of Butyrylcholinesterase? *Biochemistry* **40**, 7433–7445 (2001).
17. P. M. Lundy, M. G. Hamilton, T. W. Sawyer, J. Mikler, Comparative protective effects of HI-6 and MMB-4 against organophosphorous nerve agent poisoning. *Toxicology* **285**, 90–96 (2011).
18. N. Aurbek, H. Thiermann, L. Szinicz, P. Eyer, F. Worek, Analysis of inhibition, reactivation and aging kinetics of highly toxic organophosphorus compounds with human and pig acetylcholinesterase. *Toxicology* **224**, 91–99 (2006).
19. C. Luo, *et al.*, An *In Vitro* Comparative Study on the Reactivation of Nerve Agent-Inhibited Guinea Pig and Human Acetylcholinesterases by Oximes. *Biochemistry* **46**, 11771–11779 (2007).
20. R. Wang, H. Yan, X. Tang, Progress in studies of huperzine A, a natural cholinesterase inhibitor from Chinese herbal medicine1. *Acta Pharmacol. Sin.* **27**, 1–26 (2006).
21. J. R. Atack, Q. S. Yu, T. T. Soncrant, A. Brossi, S. I. Rapoport, Comparative inhibitory effects of various physostigmine analogs against acetyl- and butyrylcholinesterases. *J. Pharmacol. Exp. Ther.* **249**, 194–202 (1989).

22. A. J. Dafferner, *et al.*, Characterization of butyrylcholinesterase in bovine serum. *Chem. Biol. Interact.* **266**, 17–27 (2017).
23. G. Šinko, Z. Kovarik, E. Reiner, V. Simeon-Rudolf, J. Stojan, Mechanism of stereoselective interaction between butyrylcholinesterase and ethopropazine enantiomers. *Biochimie* **93**, 1797–1807 (2011).
24. M. L. Raves, *et al.*, Structure of acetylcholinesterase complexed with the nootropic alkaloid, (–)-huperzine A. *Nat. Struct. Mol. Biol.* **4**, 57–63 (1997).
25. E. Mons, S. Roet, R. Q. Kim, M. P. C. Mulder, A Comprehensive Guide for Assessing Covalent Inhibition in Enzymatic Assays Illustrated with Kinetic Simulations. *Curr. Protoc.* **2**, e419 (2022).
26. Z. Kovarik, A. Bosak, G. Šinko, T. Latas, Exploring the Active Sites of Cholinesterases by Inhibition with Bambuterol and Haloxon. *Croat. Chem. Acta* **76**, 63–67 (2003).
27. F. Worek, H. Thiermann, L. Szinicz, P. Eyer, Kinetic analysis of interactions between human acetylcholinesterase, structurally different organophosphorus compounds and oximes. *Biochem. Pharmacol.* **68**, 2237–2248 (2004).
28. F. Worek, *et al.*, Kinetic analysis of reactivation and aging of human acetylcholinesterase inhibited by different phosphoramidates. *Biochem. Pharmacol.* **73**, 1807–1817 (2007).
29. F. Worek, H. Thiermann, T. Wille, Organophosphorus compounds and oximes: a critical review. *Arch. Toxicol.* **94**, 2275–2292 (2020).
30. M. C. Chambers, *et al.*, A cross-platform toolkit for mass spectrometry and proteomics. *Nat. Biotechnol.* **30**, 918–920 (2012).
31. Å. Forsberg, G. Puu, Kinetics for the inhibition of acetylcholinesterase from the electric eel by some organophosphates and carbamates. *Eur. J. Biochem.* **140**, 153–156 (1984).
32. G. J. Hart, R. D. O’Brien, Recording spectrophotometric method for determination of dissociation and phosphorylation constants for the inhibition of acetylcholinesterase by organophosphates in the presence of substrate. *Biochemistry* **12**, 2940–2945 (1973).
33. E. I. C. Wang, P. E. Braid, Oxime Reactivation of Diethylphosphoryl Human Serum Cholinesterase. *J. Biol. Chem.* **242**, 2683–2687 (1967).
34. E. C. Meek, *et al.*, Synthesis and In Vitro and In Vivo Inhibition Potencies of Highly Relevant Nerve Agent Surrogates. *Toxicol. Sci.* **126**, 525–533 (2012).
35. R. K. Sit, *et al.*, New structural scaffolds for centrally acting oxime reactivators of phosphylated cholinesterases. *J. Biol. Chem.* **286**, 19422–19430 (2011).
36. Z. Radić, *et al.*, Refinement of structural leads for centrally acting oxime reactivators of phosphylated cholinesterases. *J. Biol. Chem.* **287**, 11798–11809 (2012).
37. B. J. Bennion, *et al.*, Development of a CNS-permeable reactivator for nerve agent exposure: an iterative, multi-disciplinary approach. *Sci. Rep.* **11**, 15567 (2021).
38. C. A. Valdez, *et al.*, Improved chemical synthesis, identification and evaluation of prospective centrally active oxime antidotes for the treatment of nerve agent exposure. *Tetrahedron* **144**, 133598 (2023).
39. J. Kalisiak, E. C. Ralph, J. Zhang, J. R. Cashman, Amidine−Oximes: Reactivators for Organophosphate Exposure. *J. Med. Chem.* **54**, 3319–3330 (2011).
